# Prioritizing monitoring and conservation efforts for fish spawning aggregations in the U.S. Gulf of Mexico

**DOI:** 10.1038/s41598-018-26898-0

**Published:** 2018-05-31

**Authors:** Arnaud Grüss, Christopher Biggs, William D. Heyman, Brad Erisman

**Affiliations:** 1 Department of Marine Biology and Ecology, Rosenstiel School of Marine and Atmospheric Science, University of Miami, 4600 Rickenbacker Causeway, Miami, FL 33149 USA; 2 University of Texas at Austin, Department of Marine Science, 750 Channel View Drive, Port Aransas, Texas 78373-5015 USA; 3 LGL Ecological Research Associates, Inc., 4103S. Texas Avenue, Bryan, TX 77802 USA

## Abstract

In the U.S. Gulf of Mexico (U.S. GOM), the identification and characterization of transient fish spawning aggregation (FSA) sites is recognized as a regional priority for conservation, but progress is hindered by a lack of understanding of FSA distributions for most exploited species. We employed information compiled in regional databases on FSAs and monitoring for the U.S. GOM to fit species distribution models and produce maps showing the areas likely to host single- and multi-species transient FSA sites. Our results revealed two distinct regions of the U.S. GOM for prioritizing monitoring and conservation efforts for transient FSAs: the coastal waters surrounding major bay systems, particularly those of Texas and Louisiana, and portions of the continental shelf edge (the Flower Garden Banks area and the West Florida shelf edge). The next step would be to locate and characterize actual transient FSA sites in the U.S. GOM by surveying within the areas we identified.

## Introduction

**Figure 1:**
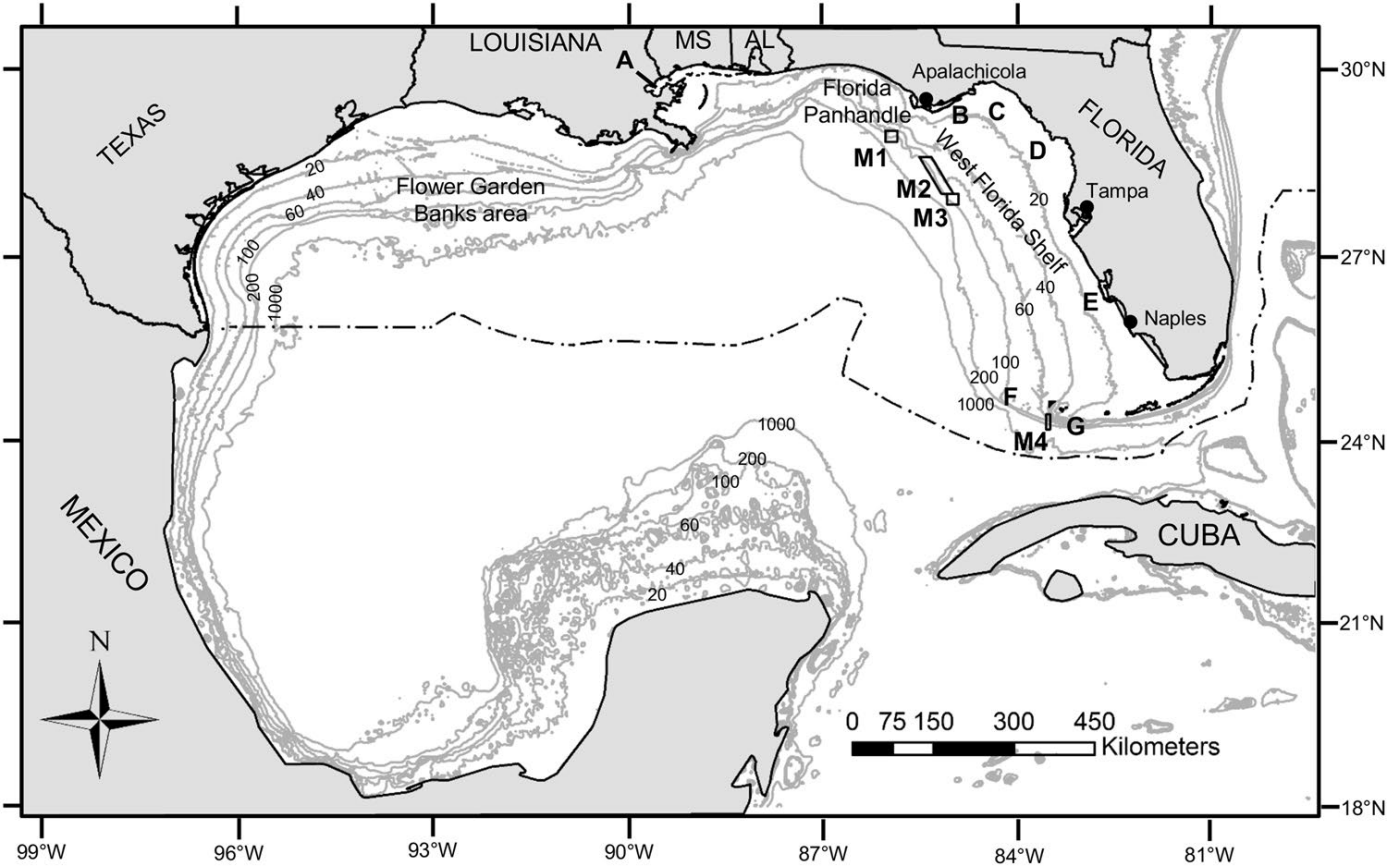
Map of the Gulf of Mexico (GOM). Depth contours are labeled in 20−, 40−, 60−, 100−, 200−, and 1000−m contours. Important features are labeled and include: the Flower Garden Banks area (i.e., the large area of submerged banks of the northwestern GOM that includes the Flower Garden Banks), the Florida Panhandle, the West Florida Shelf, Lake Pontchartrain (**A**), the Turkey Point region (**B**), the Apalachee Bay (**C**), the Cedar Key region (**D**), the Charlotte Harbor region (**E**), Pulley Ridge (**F**), and the Dry Tortugas (**G**). The spawning aggregation-based marine protected areas (MPAs) of the U.S. GOM are shown; they include: the Madison-Swanson MPA (**M1**), Steamboat Lumps (**M2**), the Edges (**M3**), and the Dry Tortugas Marine Reserve (M4). MS = Mississippi - AL = Alabama. The black dashed-dotted line delineates the U.S. exclusive economic zone. Figure produced with ArcGIS 10.4 (http://www.arcgis.com).

Fish spawning aggregations (FSAs), temporary gatherings of conspecifics for the purpose of reproduction, are gaining increased attention worldwide^1^. In the Gulf of Mexico (“GOM”), a large marine ecosystem bordered by the U.S., Mexico and Cuba (Fig. 1), FSA sites are key nodes in the life history of many species on which millions of people depend for their livelihoods^2^. FSA sites are often shared by multiple species and serve as productivity hotspots, because these small areas attract large numbers of fish to reproduce, apex predators to feed on spawning fish, and planktonic feeders to feast on masses of protein-rich eggs^3^. There are two major types of FSAs: resident FSAs and transient FSAs. Transient FSAs usually occur at a limited number of sites located outside of adult home ranges within a restricted time period, and they may be the only opportunity for participating individuals to reproduce^4^. For this reason, fishing on transient FSAs can be highly detrimental to fish population resilience^5^.

Despite their large contribution to economy and ecosystem health and their high vulnerability to fishing, transient FSA dynamics in the GOM are poorly known, and few transient FSA sites are managed or protected by any measures in the region^6–11^. Information on the biology, spatial distribution and status of transient FSAs in the GOM would aid state, federal and regional management efforts to maintain healthy, productive stocks for the benefit of fisheries and ecosystems.

Recently, the National Oceanic and Atmospheric Administration (NOAA) RESTORE Act Science Program (hereafter simply referred to as “RESTORE”) funded a synthesis project to assess existing information on FSAs, data gaps and research priorities in the GOM^8^. One major endeavor of this project was the identification of federally-managed FSA-forming species of primary concern, based on a number of criteria including, among others, the degree to which the species aggregate to spawn, their commercial and recreational landings, and their conservation status^12^. Twenty-eight species of primary concern were identified, of which 17 form transient FSAs or a mix of resident and transient FSAs (Table 1).

**Table 1:** Characteristics of the species considered in this study.

Species	Family	Reproduction mode	Total length at sexual maturity (cm)	Spawning aggregation type	Spawning months	Commercial landings (lbs)	Recreational landings (N)	Conservation status
Greater amberjack (*Seriola dumerili*)	Carangidae	Gonochoristic	79	Mixed	March to June	481954	272351	1
Almaco jack (*Seriola rivoliana*)	Carangidae	Gonochoristic	81	Mixed	April to November	36277	15341	1
Yellowedge grouper (*Hyporthodus flavolimbatus*)	Epinephelidae	Protogynous	55	Mixed	February to November	742028	656	3
Warsaw grouper (*Hyporthodus nigritus*)	Epinephelidae	Protogynous	81	Mixed	April to May	97402	943	5
Scamp (*Mycteroperca phenax*)	Epinephelidae	Protogynous	33	Mixed	January to June	246538	70454	1
Gag (*Mycteroperca microlepis*)	Epinephelidae	Protogynous	54	Transient	January to April	620534	1835929	1
Black grouper (*Mycteroperca bonaci*)	Epinephelidae	Protogynous	86	Transient	December to April	46855	5530	2
Yellowmouth grouper (*Mycteroperca interstitialis*)	Epinephelidae	Protogynous	43	Transient	January to December	421	194	3
Yellowfin grouper (*Mycteroperca venenosa*)	Epinephelidae	Protogynous	54	Transient	January to March	1511	801	1
Nassau grouper (*Epinephelus striatus*)	Epinephelidae	Protogynous	40	Transient	December to February	0	0	5
Goliath grouper (*Epinephelus itajara*)	Epinephelidae	Protogynous	120	Transient	June to October	0	3	5
Mutton snapper (*Lutjanus analis*)	Lutjanidae	Gonochoristic	50	Transient	May to August	77736	3956	1
Cubera snapper (*Lutjanus cyanopterus*)	Lutjanidae	Gonochoristic	61	Transient	June to September	1307	929	3
Black drum (*Pogonias cromis*)	Sciaenidae	Gonochoristic	65	Mixed	January to April, and August to October	0	0	1
Red drum (*Sciaenops ocellatus*)	Sciaenidae	Gonochoristic	68	Mixed	August to November	0	0	1
Sheepshead (*Archosargus probatocephalus*)	Sparidae	Gonochoristic	30	Transient	February to April	0	0	1
Southern flounder (*Paralichthys lethostigma*)	Paralichthyidae	Gonochoristic	40	Transient	October to January	0	0	2

The information provided, which was compiled in Biggs *et al*.^12^, include: reproduction mode, total length at sexual maturity (in cm), spawning aggregation type, spawning months, average commercial landings in the U.S. Gulf of Mexico (GOM) over the period 2009–2013 (in lbs), average recreational landings in the U.S. Gulf of Mexico (GOM) over the period 2009–2013 (in number of fish (N)), and status on the International Union for Conservation of Nature’s Red List of Threatened Species (1 = Least Concern, 2 = Near Threatened, 3 = Vulnerable, 4 = Endangered, 5 = Critically Endangered).

The RESTORE funded project represents a large step forward, and the next step in the GOM will be to locate transient FSA sites (particularly multi-species sites) and recommend them for protection in large enough areas to allow for the recovery and resilience of FSA-forming species^13^. However, the extensive surface area of the GOM poses a substantial issue. The U.S. GOM alone has a surface area of around 697,000 km². Therefore, there is a need for identifying the areas likely to host transient FSA sites (henceforth “potential FSA areas”) in the GOM to prioritize monitoring and conservation efforts for transient FSAs in the region.

In this study, we use the information on transient FSAs (hereafter usually called “FSAs”) compiled within the RESTORE project, and a large monitoring database compiled in previous studies^14–16^, to fit species distribution models (SDMs) and then map the distribution of potential FSA areas in the U.S. GOM. First, we employ the information on lengths at sexual maturity and spawning months compiled within the RESTORE project to extract relevant data from the large monitoring database. Then, we fit SDMs to the monitoring data and make predictions with the fitted SDMs to map the encounter probability of adult fish during the spawning season. Finally, we use the encounter probability maps for adult fish during the spawning season and a simple algorithm to produce maps showing the distribution of potential single- and multi-species FSA areas in the U.S. GOM. Here, we consider the 17 species forming transient FSAs or a mix of resident and transient FSAs identified in the RESTORE project (Table 1).

## Results

**Figure 2:**
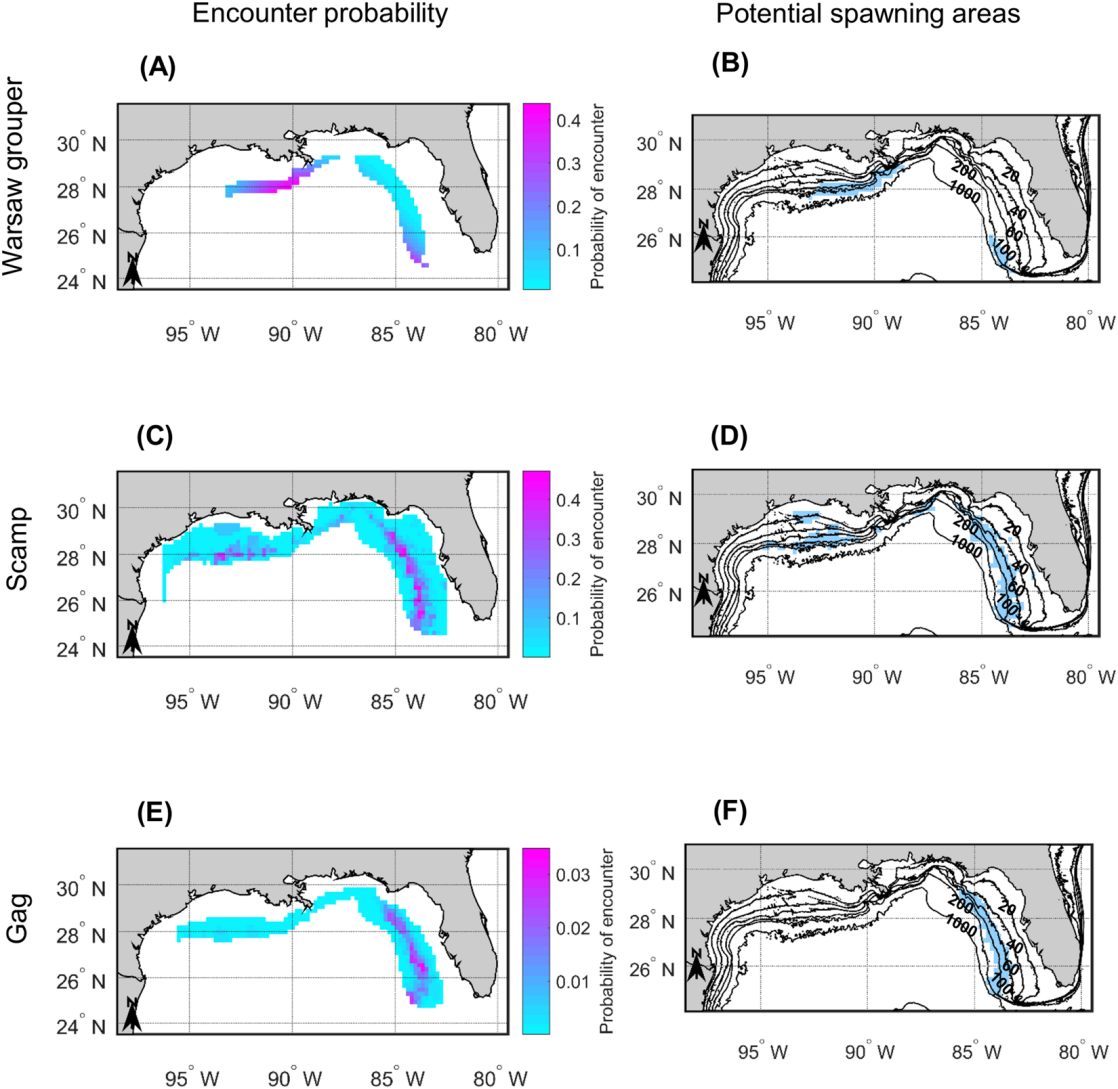
Maps showing (**A**,**C**,**E**) the encounter probability and (**B**,**D**,**F**) the potential spawning areas of three species of the U.S. Gulf of Mexico forming transient spawning aggregations. (**A**,**B**) are for Warsaw grouper (*Hyporthodus nigritus*), (**C**,**D**) are for scamp (*Mycteroperca phenax*), and (**E**,**F**) are for gag (*Mycteroperca microlepis*). The maps shown in panels (**A**,**B**) were produced from the predictions of generalized additive models accounting for spatial structure at a broad spatial scale, while the maps shown in panels (**C**,**F**) were produced from the predictions of geostatistical models. Depth contours are provided in panels (**B**,**D**,**F**); they are labeled in 20−, 40−, 60−, 100−, 200−, and 1000−m contours. Figure produced with MATLAB R2017a (https://www.mathworks.com/products/matlab.html).

We were able to fit geostatistical generalized linear mixed models (GLMMs) for four species and generalized additive models (GAMs) for eight species. The twelve SDMs passed the validation tests (Supplementary Data) and were therefore used to produce maps (Fig. 2, Supplementary Fig. S1). Due to an absence or lack of monitoring data, we were unable to fit SDMs for the following five species: yellowfin grouper (*Mycteroperca venenosa*), yellowmouth grouper (*M*. *interstitialis*), Nassau grouper (*Epinephelus striatus*), goliath grouper (*E*. *itajara*) and cubera snapper (*Lutjanus cyanopterus*) (Supplementary Table S1).

**Figure 3:**
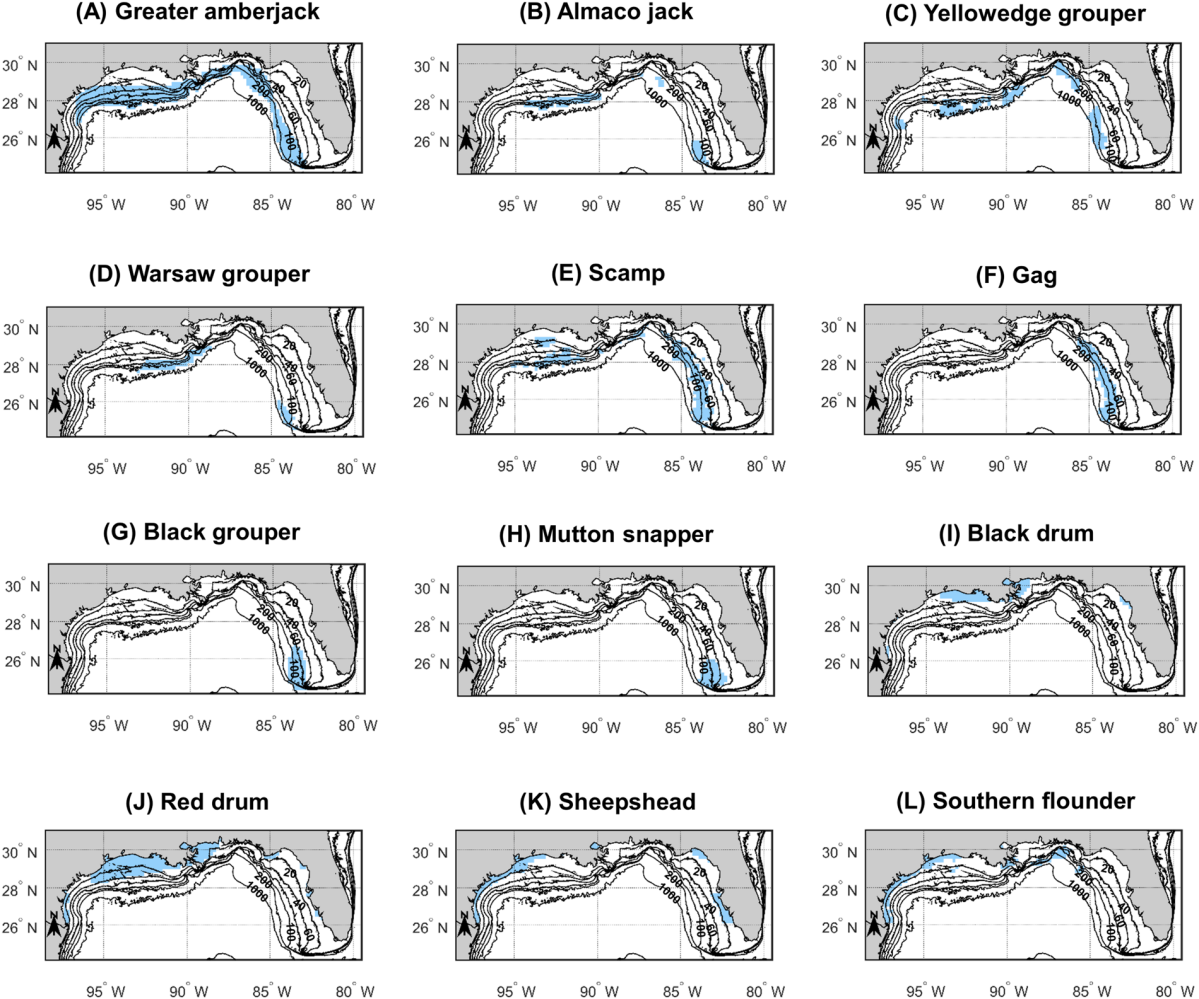
Maps showing the potential spawning areas of twelve species of the U.S. Gulf of Mexico forming transient spawning aggregations. The maps shown in panels (C, E-F, L) were produced from the predictions of geostatistical models. All the other maps were produced from the predictions of generalized additive models accounting for spatial structure at a broad spatial scale. Depth contours are provided and are labeled in 20−, 40−, 60−, 100−, 200−, and 1000−m contours. Figure produced with MATLAB R2017a (https://www.mathworks.com/products/matlab.html).

The location of potential FSA areas tends to vary from one grouper-snapper-jack species to another (Fig. 3A–H). The potential FSA areas of greater amberjack (*Seriola dumerili*) occur along the entire GOM shelf edge (Fig. 3A), while those of almaco jack (*S*. *rivoliana*) are concentrated in the Flower Garden Banks area, in Alabama and Florida Panhandle shelf waters and in the Pulley Ridge and Dry Tortugas areas (Fig. 3B). Potential FSA areas are found all over the GOM shelf for yellowedge grouper (*Hyporthodus flavolimbatus*) and scamp (*M*. *phenax*) (Fig. 3C,E). Those of Warsaw grouper (*H*. *nigritus*) are concentrated on the Louisiana shelf and in the Pulley Ridge and Dry Tortugas areas (Fig. 3D), while those of gag (*M*. *microlepis*) are concentrated on the West Florida shelf south of Apalachicola, Florida (Fig. 3F). Finally, the potential FSA areas of black grouper (*M*. *bonaci*) and mutton snapper (*Lutjanus analis*) occur on the southwestern Florida shelf (Fig. 3G–H).

Regarding the coastal species considered in this study, potential FSA areas for black drum (*Pogonias cromis*) are found in the coastal waters of Texas near the Mexican border, eastern Texas, Louisiana and Mississippi, and in the Cedar Key region (Fig. 3I). Those of red drum (*Sciaenops ocellatus*) occur in the coastal waters of all U.S. GOM states; in Florida, they are predicted to be located in the Turkey Point, Cedar Key, Tampa Bay and Charlotte Harbor regions (Fig. 3J). Potential FSA areas for sheepshead (*Archosargus probatocephalus*) are found in Texas waters, Lake Pontchartrain, the Apalachee Bay and the region between Tampa, Florida and Naples, Florida (Fig. 3K); and those of southern flounder (*Paralichthys lethostigma*) are found in Texas, Louisiana, Alabama and northwestern Florida coastal waters (Fig. 3L).

**Figure 4:**
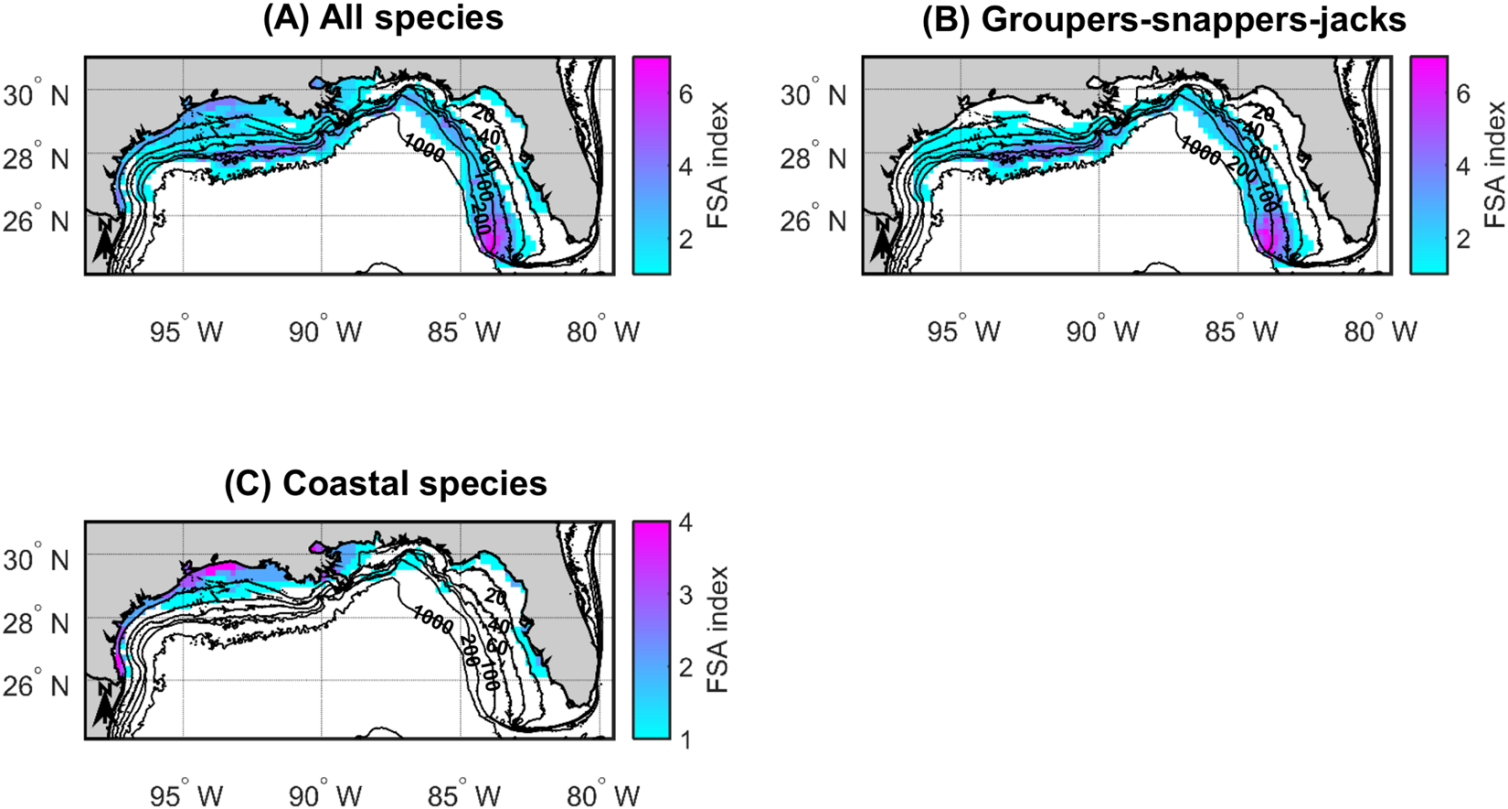
Fish spawning aggregation (FSA) indices for the U.S. Gulf of Mexico (GOM). The FSA indices shown in **(A)** were obtained by adding together the twelve maps provided in Fig. 3. The FSA indices shown in **(B)** were obtained by adding together the maps provided in Fig. 3 that were generated for groupers (Epinephelidae), snappers (Lutjanidae) and jacks (Carangidae). Finally, the FSA indices shown in **(C)** were obtained by adding together the maps provided in Fig. 3 that were generated for coastal (Sciaenidae, Sparidae and Paralichthyidae) species. Depth contours are provided and are labeled in 20−, 40−, 60−, 100−, 200−, and 1000−m contours. Figure produced with MATLAB R2017a (https://www.mathworks.com/products/matlab.html).

**Figure 5:**
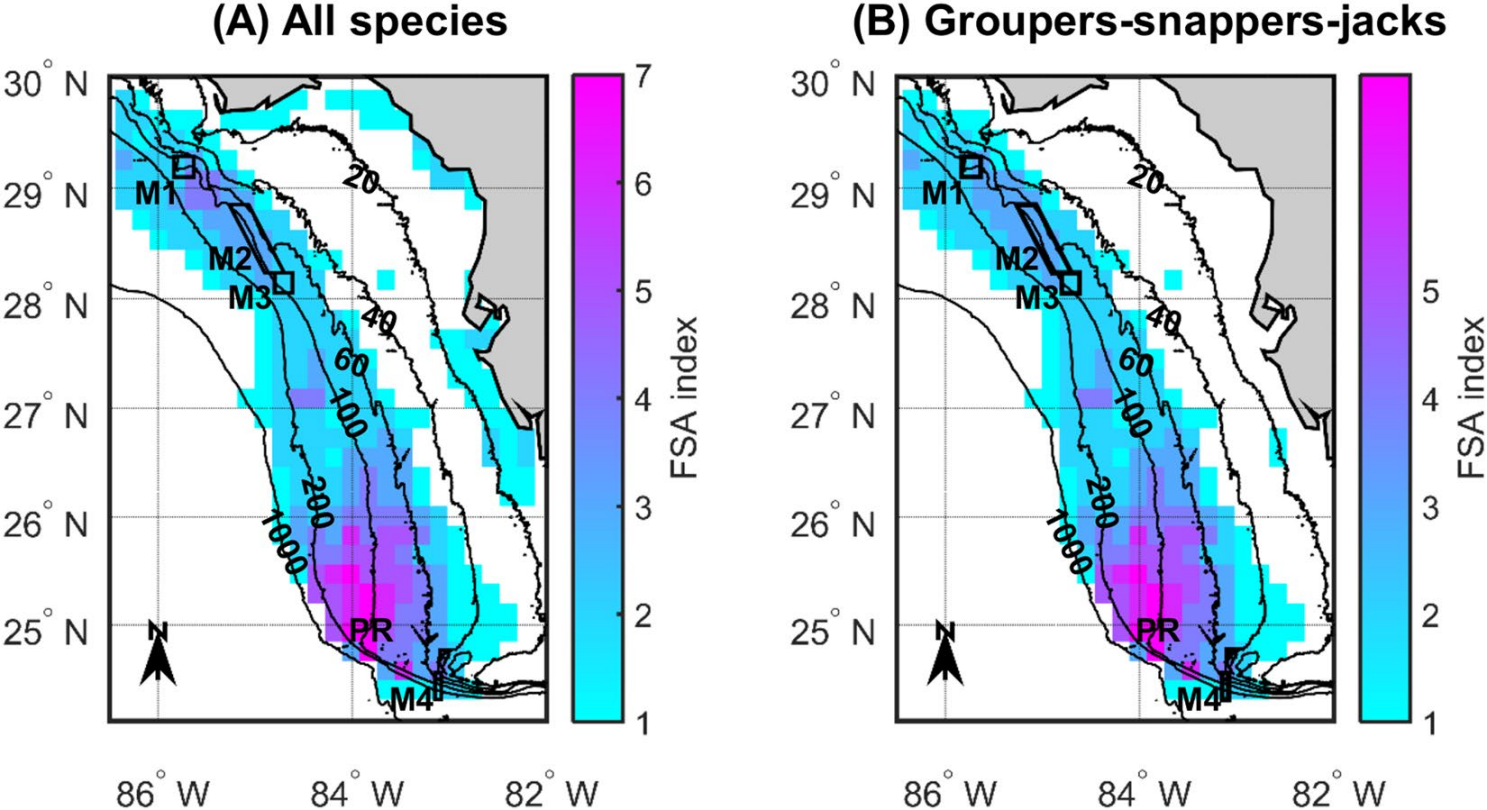
Fish spawning aggregation (FSA) indices for West Florida waters. The FSA indices shown in **(A)** were obtained by adding together the twelve maps provided in Fig. 3, while those shown in **(B)** were obtained by adding together the maps provided in Fig. 3 that were generated for groupers (Epinephelidae), snappers (Lutjanidae) and jacks (Carangidae). Depth contours are provided and are labeled in 20−, 40−, 60−, 100−, 200−, and 1000−m contours. The spawning aggregation-based marine protected areas (MPAs) implemented off West Florida are shown; they include: the Madison-Swanson MPA (M1), Steamboat Lumps (M2), the Edges (M3), and the Dry Tortugas Marine Reserve (M4). Figure produced with MATLAB R2017a (https://www.mathworks.com/products/matlab.html).

If we consider all the species for which we fitted SDMs, potential multi-species FSA areas are primarily located in coastal waters surrounding major bay systems, particularly those of Texas and Louisiana, and in the Flower Garden Banks area and along the West Florida shelf edge (Fig. 4A). The potential multi-species FSA areas of groupers-snappers-jacks occur primarily in the Flower Garden Banks area and along the West Florida shelf edge south of 26°N (Fig. 4B), while those of coastal species are primarily located in Texas and Louisiana coastal waters (Fig. 4C). Our FSA index maps suggest that existing FSA-based marine protected areas (MPAs), which are all located in West Florida waters, offer protection to multi-species FSAs (Fig. 5). However, those are not the regions of West Florida that include the potential FSA areas of the largest possible number of species. Based on our predictions, off West Florida, it would be advantageous to implement FSA-based MPAs in the Pulley Ridge region, particularly for protecting the FSAs of multiple grouper, snapper and jack species (Fig. 5). Similarly, in the western U.S. GOM, if the FSA sites of grouper, snapper and jack species could be documented in the Flower Garden Banks area, it would be beneficial to set them aside as MPAs (Fig. 4B).

## Discussion

We were able to construct maps showing areas likely to host the FSA sites (the potential FSA areas) of 12 of the 17 study species. Our maps revealed the existence of two groups of species forming transient FSAs in the U.S. GOM: (1) a group made up of the coastal (Sciaenidae, Sparidae and Paralichthyidae) species, which spawns in coastal waters, particularly those surrounding major bay systems (Figs. 4C); and (2) a group consisting of the groupers, snappers and jacks, which reproduces offshore (Fig. 4B). Consequently, potential multi-species FSA areas in the U.S. GOM are located both in coastal waters surrounding major bay systems, particularly those of Texas and Louisiana, and in offshore regions associated with the continental shelf edge (the Flower Garden Banks area and the West Florida shelf edge). Our predictions concur with the findings of a paper in preparation, where FSA locations in the U.S. GOM are being mapped from the existing literature, data derived from historical histology collections, reliable accounts and personal observations from fishers’ logbooks and data collected by some of the authors of the present study^17^. Moreover, the spatial distribution patterns predicted for the U.S. GOM in the present study are congruent with those reported in adjacent marine regions. For example, the major channel passes along the coasts of Georgia, North Carolina and South Carolina are known to harbor FSAs of red drum, black drum, southern flounder and sheepshead at different times of the year^18–23^. Similarly, numerous species of groupers (e.g. Nassau grouper, black grouper, yellowfin grouper), snappers (e.g. cubera snapper, mutton snapper) and jacks (e.g. greater amberjack, almaco jack) co-occur at multi-species FSA sites in the Caribbean along the continental shelf edge^24–26^.

We were unable to produce maps showing the potential FSA areas of five study species: yellowfin, Nassau, yellowmouth and goliath groupers, and cubera snapper. We were not surprised to be unable to generate maps for Nassau grouper, which is absent from the U.S. waters of the GOM^27^, and for yellowfin grouper, which occurs only in a few locations of the U.S. GOM^28^.

We did not have enough data to fit SDMs and produce maps for cubera snapper and goliath grouper. Yet, there are anecdotal reports of cubera snapper forming FSAs off Texas, Louisiana and Florida^13^. Regarding goliath grouper, the species is relatively frequently encountered by the Reef Environmental Education Foundation (REEF) Fish Survey, a monitoring program based on the visual observations of volunteer divers^29^; however, the REEF Fish Survey does not measure fish lengths and was therefore not considered in this study. Recent reports suggest that goliath grouper is present throughout the U.S. GOM^13,30^. Monitoring efforts targeting cubera snapper and goliath grouper throughout the U.S. GOM should be initiated. This is particularly critical in the case of goliath grouper to improve our knowledge of the spatial distribution and ecology of the species, because the species has long been listed as “critically endangered” (by the International Union for Conservation of Nature and the U.S. Endangered Species Act), but there have been discussions about reopening its fishery^31^.

It was surprising not to have enough data to fit a SDM and produce maps for yellowmouth grouper, given that the species may be common in the western U.S. GOM^32^. We suspect that this lack of data may be because yellowmouth groupers are often misidentified as scamps^13^. Consequently, Fig. 2D actually shows the potential FSA areas of scamp and/or yellowmouth in the U.S. GOM. In the future, efforts should be made to clearly distinguish between the yellowmouth groupers and scamps that are sampled and landed to improve the assessment and management of the two species.

It is important to emphasize that, because transient FSAs are generally short-lived events, some of the areas identified in our maps may not host FSA sites, but may rather be areas where spawners feed, rest or occupy cleaning stations before or between spawning events^3^, or adult home ranges because evidence suggests that, in many fish, some adults skip spawning^33,34^. Yet, even if our study determined only areas likely to host transient FSA sites, it provides valuable information for prioritizing validation and monitoring efforts for transient FSAs; we identified a relatively small number of 20 km × 20 km areas of the vast U.S. GOM region that are likely to host FSA sites for 12 species of primary concern or multi-species FSA sites.

Conservation efforts, especially in vast regions such as the U.S. GOM, require projects synthesizing large amounts of information and projects developing management support tools, but also interactions between these two types of projects. In this study, we employed the information compiled by empiricists for a synthesis project and the large monitoring database developed by modelers for another project, to locate potential single- and multi-species FSA areas in the U.S. GOM. The next step would be to locate and verify actual FSA sites in the U.S. GOM, by surveying within the 20 km × 20 km areas we identified during the spawning seasons of the species of interest. This endeavor, which should involve fishers, would consist of locating candidate FSA sites within the 20 km × 20 km areas based on fishers’ interviews, satellite images, aerial photographs and bathymetric charts; and then collecting evidence of spawning at the sites, such as videos of gamete release or females with hydrated oocytes^35^. Ultimately, it will be possible to establish well-informed spatial management plans to better protect transient FSAs from overfishing in the U.S. GOM. Our results suggest that a priority is to survey the Pulley Ridge and Flower Garden Banks areas to locate and verify the actual FSA sites of groupers, snappers and jacks in these areas and set these FSA sites aside as MPAs (Figs 4 and 5). Achieving ambitious conservation objectives requires that empiricists, modelers, fishers and resource managers all work closely together^2,35^.

## Methods

### Study species

The 17 species considered in this study include two jacks (Carangidae), nine groupers (Epinephelidae), two snappers (Lutjanidae), two Sciaenidae, one Sparidae, and one Paralichthyidae (Table 1). The study groupers are all protogynous, i.e., mature first as females and then change into males, while all the other species considered in this study are gonochoristic. Among the grouper species, gag (*Mycteroperca microlepis*), is relatively unique in that the adult females and males of the species are spatially segregated during most of the year; adult male gags stay at FSA sites year-round and are joined by adult females during the spawning season^7,36^. Thus, to identify gag potential FSA areas, it is more relevant to determine the annual spatial distribution of adult male gags than the spatial distribution of the entire adult gag population during the spawning season^37^.

### Monitoring data

Previous studies^14–16^ compiled a large monitoring database gathering all the monitoring data collected in the U.S. GOM over the period 2000–2016 using random sampling methods. For this study, we employed the 26 fisheries-independent and eight fisheries-dependent datasets from the large monitoring database for the U.S. GOM that collated length information (Table 2, Supplementary Table S2). From each dataset, we extracted the following information: (1) the longitudes and latitudes at which monitoring took place; (2) the years and months during which monitoring took place; and (3) whether the adults (all species but gag) or adult males (gag) of the study species were encountered or not during sampling events. Encounters/non-encounters for adult fish were obtained using the lengths collated during monitoring events and the lengths at sexual maturity compiled in the RESTORE project^12^ (Table 1). Encounters/non-encounters for adult male gag were extracted using the lengths collated during monitoring events and gag length at sex change (102 cm TL^33^).

**Table 2:** Fisheries-independent and fisheries-dependent datasets from the large monitoring database for the U.S. Gulf of Mexico considered in this study.

Name of the monitoring program	Alias
Alabama Marine Resources Division (AMRD) Fisheries Assessment and Monitoring Program (FAMP) Gillnet Survey (fisheries-independent)	ALGILL
National Marine Fisheries Service (NMFS) Bottom Longline Survey (fisheries-independent)	BLL
Deep Pelagic Nekton Dynamics of the Gulf of Mexico (DEEPEND) Survey (fisheries-independent)	DEEPEND
NMFS Expanded Annual Stock Assessment (EASA) Survey – Longline (fisheries-independent)	EASALL
NMFS EASA Survey – Vertical Line (fisheries-independent)	EASAVL
Fish and Wildlife Research Institute (FWRI) Bay Seine Survey (fisheries-independent)	FLBAY
FWRI Haul Seine Survey (fisheries-independent)	FLHAUL
FWRI For-Hire At-Sea Observer Program (fisheries-dependent)	FLOBS
FWRI Purse Seine Survey (fisheries-independent)	FLPURSE
FWRI Reef Fish Trap Survey (fisheries-independent)	FLTRAP
FWRI Trawl Survey (fisheries-independent)	FLTRAWL
Gulf of Mexico Fisheries Information Network (GulfFIN) Head Boat Port Sampling Program (fisheries-dependent)	GULFFINPORT
NMFS Gulf of Mexico Shark Pupping and Nursery (GULFSPAN) Survey (fisheries-independent)	GULFSPAN
Southeast Area Monitoring and Assessment Program (SEAMAP) Gulf of Mexico Inshore Bottom Longline Survey (fisheries-independent)	INBLL
Louisiana Department of Wildlife and Fisheries (LDWF) Vertical Line Survey (fisheries-independent)	LAVL
Mississippi Department of Marine Resources (MDMR) Sport Fish Shark Gillnet Survey (fisheries-independent)	MSGILL
MDMR Sport Fish Shark Handline Survey (fisheries-independent)	MSHAND
MDMR Fisheries Assessment and Monitoring (FAM) Trawl Survey (fisheries-independent)	MSTRAWL
NMFS Southeast Gillnet Observer Program (fisheries-dependent)	OBSGILL
Reef Fish Bottom Longline Observer Program (fisheries-dependent)	OBSLL
Southeastern Shrimp Fisheries Observer Coverage Program (fisheries-dependent)	OBSSHRIMP
Reef Fish Vertical Line Observer Program (fisheries-dependent)	OBSVL
NMFS Panama City Trap Survey (fisheries-independent)	PCTRAP
NMFS Panama City Video Survey (fisheries-independent)	PCVIDEO
NMFS Pelagic Observer Program (fisheries-dependent)	POP
NMFS Shark Bottom Longline Observer Program (fisheries-dependent)	SBLOP
NMFS Small Pelagics Survey (fisheries-independent)	SMALLPEL
SEAMAP Groundfish/Trawl Survey (fisheries-independent)	TRAWL
Texas Parks and Wildlife Department (TPWD) Bottom Longline Survey (fisheries-independent)	TXBLL
TPWD Gillnet Survey (fisheries-independent)	TXGILL
TPWD Seine Survey (fisheries-independent)	TXSEINE
TPWD Trawl Survey (fisheries-independent)	TXTRAWL
SEAMAP Reef Fish Video Survey (fisheries-independent)	VIDEO
SEAMAP Gulf of Mexico Vertical Longline Survey (fisheries-independent)	VL

Details about the datasets can be found in Supplementary Table S2.

For each species, we established which monitoring datasets, years and months should be considered to fit SDMs. To select monitoring datasets and years for a given species, we applied, to the extent possible, the following rules: (1) monitoring datasets with fewer than 20–50 encounters were excluded, following the recommendations of Leathwick *et al*.^38^ and Austin^39^; and (2) years with fewer than five encounters were excluded. The latter rule was established in recent studies that fitted SDMs to the large monitoring database for the U.S. GOM for generating products for ecosystem models^14–16^. For all study species except gag, only monitoring data collected during the spawning months of the species (Table 1) were considered to fit SDMs; in the case of gag, monitoring data collected during any month of the year were considered. The monitoring data employed in this study and the products derived from them are not available to readers, because they include fisheries-dependent data that are confidential.

### Statistical modeling

We proceeded in two steps. First, for each species, we tried to fit a geostatistical binomial GLMM^15,37^ to monitoring data. Geostatistical binomial models are based on the tenet that encounter probability at a given location resembles more encounter probability at neighboring locations than encounter probability at remote sites, i.e., these models account for spatial structure at a fine spatial scale. Thus, geostatistical binomial GLMMs estimate a smoothed surface that accurately describes how encounter probability varies over space^40^. Second, for those species for which we were unable to fit a geostatistical GLMM (because the model did not converge due to a lack of encounter estimates), we fitted a binomial GAM accounting for spatial structure at a broad spatial scale (through the integration of an interaction term between eastings and northings)^14^ to monitoring data.

Briefly, our geostatistical binomial GLMMs integrate Gaussian Markov random fields to model spatial residuals in encounter probability, as well as the fixed effect of year and the random effect of monitoring program^15,37^. Template Model Builder called within the R environment^41^ was used to estimate geostatistical GLMM parameters (Supplementary Methods).

In addition to the interaction term between eastings and northings, our binomial GAMs integrate the fixed effects of monitoring program and year^14^. GAMs were fit in the R environment, using the “mgcv” package^42^ (Supplementary Methods).

### Mapping

We mapped the encounter probability and the hotspots of adults of the study species during the spawning season. First, to be able to generate encounter probability maps, we produced prediction grids for the study species from a 20 km × 20 km spatial grid covering the whole U.S. GOM. The prediction grids were constructed based on the ranges of latitude, longitude and depth at which adults of the study species are encountered by monitoring programs during the spawning season (or year-round in the case of gag). To determine depth ranges, we relied on a 20 km × 20 km depth raster generated from the SRTM30 PLUS global bathymetry grid from the GOM Coastal Ocean Observing System^43^.

Second, for the species for which it was possible to fit a geostatistical GLMM, we employed the fitted GLMM and the prediction grid for the species to produce an encounter probability map for each of the sampling years. Then, the encounter probability maps for each sampling year were averaged to generate one long-term encounter probability map^15,37^. For the species for which it was only possible to fit a GAM, we produced long-term encounter probability maps using the fitted GAM and the prediction grid for the species, and the average year effect and the monitoring program effect with the highest selectivity^14,44,45^.

Third, we constructed hotspot maps from the long-term encounter probability maps. The hotspots of a given species during the spawning season (year-round in the case of gag) are the cells of its prediction grid where, during the spawning season (year-round in the case of gag), the encounter probability the species is equal to or greater than the mean encounter probability of the species over the entire prediction grid^15,46,47^. The hotspot map generated for a given species indicate the location of its potential FSA areas.

We also produced “FSA index maps” by adding together hotspot maps for individual species^14^; the higher the FSA index in a given area, the higher the chances that multi-species FSAs occur in that area. The first FSA index map was created by adding together hotspot maps for all the study species. The second FSA index map was constructed from the hotspot maps generated for groupers, snappers and jacks. Groupers and snappers are the FSA-forming species that usually receive the most attention^1^. While the timing of spawning for jacks may differ from that for groupers and snappers, both greater amberjack (*Seriola dumerili*) and almaco jack (*S*. *rivoliana*) have been documented to aggregate and spawn at the same sites as groupers in numerous locations in the Caribbean^25,26^. Finally, the third hotspot index map was constructed from the hotspot maps generated for coastal species, namely the Sciaenidae, Sparidae and Paralichthyidae species considered in this study.

To gauge the degree of protection offered by existing FSA-based marine protected areas (MPAs), we overlaid FSA index maps with the boundaries of these MPAs. At present, four FSA-based MPAs are implemented in West Florida waters: the Madison-Swanson MPA, Steamboat Lumps, the Edges, and the Dry Tortugas Marine Reserve (Fig. 1). The Madison-Swanson MPA, Steamboat Lumps and the Edges are partial-take year-round MPAs, where only recreational pelagic trolling activities are allowed during a fraction of the year^7,9^; these MPAs were established to protect the FSAs of reef fish species, particularly gag and scamp. The Dry Tortugas Marine Reserve is a no-take year-round MPA, which was created in 2001 in part to protect the FSAs of mutton snapper^10,11,48^.

## Electronic supplementary material

Supplementary Information
