# Author Correction: Prioritizing monitoring and conservation efforts for fish spawning aggregations in the U.S. Gulf of Mexico

**DOI:** 10.1038/s41598-018-28120-7

**Published:** 2018-06-26

**Authors:** Arnaud Grüss, Christopher Biggs, William D. Heyman, Brad Erisman

**Affiliations:** 1 Department of Marine Biology and Ecology, Rosenstiel School of Marine and Atmospheric Science, University of Miami, 4600 Rickenbacker Causeway, Miami, FL 33149 USA; 2 University of Texas at Austin, Department of Marine Science, 750 Channel View Drive, Port Aransas, Texas 78373-5015 USA; 3 LGL Ecological Research Associates, Inc., 4103S. Texas Avenue, Bryan, TX 77802 USA

Correction to: *Scientific Reports* 10.1038/s41598-018-26898-0, published online 31 May 2018

This Article contains an error in Table 1.

In the ‘Reproduction mode’ column, Nassau grouper (Epinephelus striatus) should be listed as ‘Gonochoristic’, not ‘Protogynous’.

