# Supplementary material for: Prioritizing monitoring and conservation efforts for fish spawning aggregations in the U.S. Gulf of Mexico
Source: Sci Rep. 2018 May 31;8:8473. doi: 10.1038/s41598-018-26898-0 (PMC5981459; doi:10.1038/s41598-018-26898-0)

**Prioritizing monitoring and conservation efforts for fish spawning aggregations in the U.S. Gulf of Mexico**

**Supplementary Information**

Arnaud Grüss, Christopher Biggs, William D. Heyman & Brad Erisman

**Supplementary Methods. Details of the statistical modeling efforts conducted in the present study.**

We proceeded in two steps. First, for each of the study species, we tried to fit a geostatistical binomial generalized linear mixed model (GLMM)^1,2^ to monitoring data. Geostatistical binomial models are based on the tenet that encounter probability at a given location resembles more encounter probability at neighboring locations than encounter probability at remote sites, i.e., these models account for spatial structure at a fine spatial scale. Thus, geostatistical binomial GLMMs estimate a smoothed surface accurately describing how encounter probability varies over space^3^. Second, for those species for which we were unable to fit a geostatistical GLMM (because the model did not converge due to a lack of data), we fitted a binomial generalized additive model (GAM) accounting for spatial structure at a broad spatial scale (through the integration of an interaction term between eastings and northings) to monitoring data^4^.

**Geostatistical GLMMs**

The geostatistical GLMMs employed in the present study are spatio-temporal binomial GLMMs which predict encounter probabilities, and Gaussian Markov random fields are used to model spatial residuals in encounter probability^1,2^. We approximated Gaussian Markov random fields using 1000 “knots”, for computational efficiency^3^; for each study species, the location of the knots was determined through the application of a *k-*means algorithm to the locations of the data of the large monitoring database for the U.S. Gulf of Mexico (GOM), which distributes knots over space after having considered the sampling intensity of the different monitoring programs retained for a given species.

We fitted our geostatistical binomial GLMMs to the data from the large monitoring database for the U.S. GOM following the equation:

| $g\left( p_{i} \right)=\sum_{t=1}^{n_{t}} \beta_{t}Y_{i,t}+\sum_{m=1}^{n_{m}} \gamma_{m}G_{i,m}+\varepsilon_{J(i)}$ | (1) |
| --- | --- |

where *p_i_* is the encounter probability at site *s(i)*; *g* is the logit link function between *p_i_* and each term provided at the right side of the equation; $\varepsilon_{J(i)}$ are the effects of the spatial residuals in encounter probability at *J(i)*, the nearest knot to sample *i*, on the logit scale; $\sum_{m=1}^{n_{m}} \gamma_{m}G_{i,m}$ is the effect of monitoring program on *p_i_* on the logit scale; and $\sum_{t=1}^{n_{t}} \beta_{t}Y_{i,t}$ is the effect of year on *p_i_* on the logit scale. The terms $\varepsilon_{J(i)}$ are random effects, the effect of monitoring program is treated as random via the implementation of Restricted Maximum Likelihood (REML), and the year effect is fixed.

As regards the effect of monitoring program, $G_{i,m}$ is a design matrix such that $G_{i,m}$is one for the monitoring program $m$ which collected sample *i* and zero otherwise; $\gamma_{m}$is a monitoring program effect (where $\gamma_{m}=0$ for the monitoring program $m$ with the largest sample size for a given species; we imposed this constraint for identifiability of all year effects $\beta_{t}$); and $n_{m}$ is the number of monitoring programs retained for the species considered.

As regards the effect of year, $Y_{i,t}$ is a design matrix such that $Y_{i,t}$ is one for the year $t$ during which sample *i* was collected and zero otherwise; $\beta_{t}$ is an intercept varying among years; and $n_{t}$ is the number of sampling years for the species considered. To predict probability of encounter in any site *i*, the geostatistical GLMMs employ data in every year $t$. Then, the intercept term $\beta_{t}$ serves to scale probability of encounter up or down amongst years, where the change in probability of encounter (in the logit scale) between years is the same for any location. Thus, $\beta_{t}$ takes into account the fact that different years may have a lower or higher probability of encounter for all locations in a given year. Then, if the spatial extent of a given program is altered amongst years, the geostatistical GLMMs takes this into account by comparing it with the predicted probability of encounter at each location.

As regards the random effects $\varepsilon_{J(i)}$, those follow a multivariate normal distribution:

| $\boldsymbol{\varepsilon}\sim MN\left( \boldsymbol{0},\boldsymbol{\Sigma} \right)$ | (2) |
| --- | --- |

where$MN$ is the multivariate normal distribution, whose expected value was fixed to 0 for each site; and $\boldsymbol{\Sigma}$ is a covariance matrix for $\boldsymbol{\varepsilon}$ at each site. We assumed that the covariance between sites *s* and *s’* is stationary and follows a Matérn distribution with smoothness *ν* = 1:

| $\Sigma\left( s,s^{'} \right)=\sigma_{\varepsilon}^{2}.Matérn\left( \left\Vert\mathbf{H}\left( s-s' \right) \right\Vert;\kappa\right)$ | (3) |
| --- | --- |

where $\sigma_{\varepsilon}$ is the standard deviation of $\boldsymbol{\varepsilon}$; **H** is the linear transformation representing geometric anisotropy; $\left( s-s' \right)=\left( x-x^{'},y-y^{'} \right)$ is the difference in eastings and northings between sites *s* and *s’*; $\left\| \mathbf{H}\left( s-s' \right) \right\|$ is the distance between sites after having accounted for geometric anisotropy^3,5^; and *κ* is the range parameter, which governs the distance over which covariance reaches 10% of its pointwise value^6^.

To estimate the fixed effect of year, we employed maximum marginal likelihood while integrating across the random effects of gear and $\boldsymbol{\varepsilon}$; the Laplace approximation implemented in the Template Model Builder^7^ was used to approximate maximum marginal likelihood. More precisely, we first approximated the probability of the random effects through use of the stochastic partial differential equation approximation for Gaussian random fields with geometric anisotropy^3^. Then, we maximized the marginal likelihood through conventional non-linear optimization in the R environment.

To determine whether the geostatistical binomial GLMMs fitted for the different species converged, we examined whether any of the parameters *H*, κ and *σ_ε_* hit an upper or lower bound, and whether the absolute value of the final gradient for each of these parameters was close to zero.

To gauge GLMM fits, we calculated Pearson residuals for a given set of samples, $i\in S$. To do so, we calculated the expected number of encounters $\hat{c}_{S}$ for that set: $\hat{c}_{S}=\sum_{i\in S} p_{i}$; and we also calculated the observed number of encounters $c_{S}$ for that set: $c_{S}=\sum_{i\in S} b_{i}$, where $b_{i}$ is the observation for sample $i$ (i.e., $b_{i}=1$ if the sample observed a given species and zero otherwise). We then calculated the Pearson residual for set $S$ as:

| $d_{S}=\frac{c_{S}-\hat{c}_{S}}{\sqrt{\frac{\hat{c}_{S}(\left\vert S \right\vert-\hat{c}_{S})}{\vert S\vert}}}$ | (4) |
| --- | --- |

where $|S|$ is the number of samples within set $S$, and the denominator is the expected standard deviation of encounters. Next, we calculated Pearson residuals $d_{S}$ for two types of analysis:

1. *Bin by predicted encounter probability*: We defined a set of samples $S$ where $0<p_{i}<0.05$, and calculated Pearson residual $d_{0<p_{i}<0.05}$. We then repeated for every bin with 0.05 width from 0.0 to 1.0.
2. *Bin by knot*: We defined a set of samples $S$ associated with a given knot $J$, and calculated the Pearson residual for that knot, $d_{J}$. We then repeated for every knot, and plotted Pearson residuals on a map.

For each of the study species, we considered the first type of residuals to identify whether the GLMM was able to predict samples with high or low expected encounter probabilities. If this was not the case, we also considered the second type of residuals to determine whether there are areas in the U.S. GOM in which the GLMM systematically under or over-estimates encounter probability

To generate the probability of encounter maps for the U.S. GOM for each of the study species, we made the assumption that the Gaussian random field in each cell of their prediction grid is equal to the value of the Gaussian random field at the nearest knot.

**GAMs**

For each of the study species for which it was not possible to fit a geostatistical GLMM, we fitted a binomial GAM using data from the large monitoring database for the U.S. GOM and the R package “mgcv”^8^:

| $g(\eta)=te(X, Y)+factor(year)+factor(monitoring program)$ | (5) |
| --- | --- |

where *η* is the probability of encounter; *g* is the logit link function between *η* and each predictor; and $te(X,Y)$ is a tensor product smooth fitted to eastings and northings. Monitoring program and year are “nuisance” variables treated as fixed effect factors^4,9,10^. We could have developed generalized additive mixed models (GAMMs)^11^ treating monitoring program as a random effect rather than GAMs. We did not choose this option, because GAMMs are computationally intensive and are likely to face convergence issues when fitted to large monitoring datasets like ours. However, Grüss *et al.*^4^ showed that the spatial patterns of probability of encounter predicted by GAMs treating monitoring program as a fixed effect factor are unaffected by the monitoring program factor, and that the gross magnitude of the probabilities of encounter predicted by these GAMs are only slightly affected by the monitoring program factor.

To evaluate the validity of the fitted GAMs, we used the “Leave Group Out Cross Validation” procedure^12,13^. In this iterative cross-validation procedure, for each of the species for which we fitted a GAM, monitoring data were randomly split into training and test datasets, with 60% of the data going to the training dataset and the rest of the data to the test dataset^14^. We fitted binomial GAMs to the training dataset employing the fitting procedure described in Equation 5, and then evaluated the GAMs using the test dataset. We repeated this process 10 times, i.e., for each individual binomial GAM, 10 models were fitted to training datasets and then evaluated using the test datasets corresponding to the training datasets.

Two metrics were utilized to evaluate binomial GAMs through the Leave Group Out Cross Validation procedure: (1) the area under the receiver operating characteristic (ROC) curve (AUC), which reflects if binomial GAMs are able to discriminate between encounters and non-encounters^15^; and (2) the adjusted coefficient of determination (adjusted *R*^2^), which is a means to measure the proportion of variance of the encounter probability explained by binomial GAMs^16^. An AUC value of 0.5 indicates no improvement in predictability over random chance, while an AUC value of 1 is indicative of perfect discrimination between encounters and non-encounters^17–19^. AUC values larger than 0.9 are indicative of excellent discrimination between encounters and non-encounters, while AUC values between 0.7 and 0.9 indicate that the binomial GAM is able to reasonably discriminate between encounters and non-encounters^20–22^. The R package “pROC” was employed to construct ROC curves and compute AUCs^23^.

For each of the species for which we fitted a GAM, we considered that their GAM passed the validation test if: (1) the median AUC value estimated via the Leave Group Out Cross Validation procedure was larger than 0.7^17–19^; and (2) the median adjusted *R*^2^ estimated via the Leave Group Out Cross Validation procedure was larger than 0.1^16^.

**References of the Supplementary Methods**

1. Grüss, A., Thorson, J. T., Babcock, E. A. & Tarnecki, J. H. Producing distribution maps for informing ecosystem-based fisheries management using a comprehensive survey database and spatio-temporal models. *ICES J. Mar. Sci.* **75,** 158-177 (2018).

2. Grüss, A. *et al.* Ontogenetic spatial distributions of red grouper (*Epinephelus morio*) and gag grouper (*Mycteroperca microlepis*) in the US Gulf of Mexico. *Fish. Res.* **193,** 129–142 (2017).

3. Thorson, J. T., Shelton, A. O., Ward, E. J. & Skaug, H. J. Geostatistical delta-generalized linear mixed models improve precision for estimated abundance indices for West Coast groundfishes. *ICES J. Mar. Sci.* **72,** 1297–1310 (2015).

4. Grüss, A., Chagaris, D. D., Babcock, E. A. & Tarnecki, J. H. Assisting ecosystem-based fisheries management efforts using a comprehensive survey database, a large environmental database and generalized additive models. *Mar. Coast. Fish.* **10,** 40-70 (2018).

5. Cressie, N. & Wikle, C. K. *Statistics for spatio-temporal data* (John Wiley & Sons, 2015).

6. Thorson, J. T., Fonner, R., Haltuch, M. A., Ono, K. & Winker, H. Accounting for spatiotemporal variation and fisher targeting when estimating abundance from multispecies fishery data. *Can. J. Fish. Aquat. Sci.* **73,** 1–14 (2016).

7. Kristensen, K., Nielsen, A., Berg, C. W., Skaug, H. & Bell, B. TMB: automatic differentiation and Laplace approximation. *J. Stat. Softw.* **70,** 1–21 (2016).

8. Wood, S. N. *Generalized additive models: an introduction with R* (CRC press, 2006).

9. Farmer, N. A. & Karnauskas, M. Spatial distribution and conservation of speckled hind and warsaw grouper in the Atlantic Ocean off the southeastern US. *PloS One* **8,** e78682 (2013).

10. Grüss, A. *et al.* Improving the spatial allocation of functional group biomasses in spatially-explicit ecosystem models: insights from three Gulf of Mexico models. *B. Mar. Sci.* **92,** 473–496 (2016).

11. Lin, X. & Zhang, D. Inference in generalized additive mixed modelsby using smoothing splines. *J. R. Stat. Soc. Series B Stat. Methodol.* **61,** 381–400 (1999).

11. Hastie, T., Tibshirani, R. & Friedman, J. *The Elements of Statistical Learning: Data Mining, Inference, and Prediction* (Springer-Verlag, 2001).

12. Kuhn, M. & Johnson, K. *Applied Predictive Modeling* (Springer Science & Business Media, 2013).

13. Grüss, A., Yemane, D. & Fairweather, T. P. Exploring the spatial distribution patterns of South African Cape hakes using generalised additive models. *Afr. J. Mar. Sci.* **38,** 395–409 (2016).

15. Hanley, J. A. & McNeil, B. J. The meaning and use of the area under a receiver operating characteristic (ROC) curve. *Radiology* **143,** 29–36 (1982).

15. Legendre, P. & Legendre, L. *Numerical Ecology (second English ed.)* (Elsevier Science, 1998).

16. Heinänen, S., Rönkä, M. & Von Numers, M. Modelling the occurrence and abundance of a colonial species, the arctic tern *Sterna paradisaea* in the archipelago of SW Finland. *Ecography* **31,** 601–611 (2008).

17. Leathwick, J. R., Elith, J. & Hastie, T. Comparative performance of generalized additive models and multivariate adaptive regression splines for statistical modelling of species distributions. *Ecol. Model.* **199,** 188–196 (2006).

18. Wintle, B. A., Elith, J. & Potts, J. M. Fauna habitat modelling and mapping: a review and case study in the Lower Hunter Central Coast region of NSW. *Austral Ecol.* **30,** 719–738 (2005).

20. Swets, J. A. Measuring the accuracy of diagnostic systems. *Science* **240,** 1285–1293 (1988).

20. Pearce, J. & Ferrier, S. Evaluating the predictive performance of habitat models developed using logistic regression. *Ecol. Model.* **133,** 225–245 (2000).

21. Fielding, A. H. & Bell, J. F. A review of methods for the assessment of prediction errors in conservation presence/absence models. *Environ. Conserv.* **24,** 38–49 (1997).

22. Robin, X. *et al.* pROC: an open-source package for R and S+ to analyze and compare ROC curves. *BMC Bioinformatics* **12,** 77 (2011).

**Supplementary Data. Validation of the geostatistical generalized linear mixed models (GLMMs) and generalized additive models (GAMs) fitted in the present study.**

We fitted a geostatistical binomial GLMM for four of the study species: (1) yellowedge grouper (*Hyporthodus flavolimbatus*); (2) scamp (*Mycteroperca phenax*); (3) gag (*Mycteroperca microlepis*); and (4) southern flounder (*Paralichthys lethostigma*). For these four species, we found that none of the parameters *H*, κ and *σ_ε_* hit an upper or lower bound, and that the absolute value of the final gradient for each of these parameters was smaller than 3.10^-4^ (Table 1). Thus, there was no evidence of non-convergence for any of the four species for which a geostatistical GLMM was fitted. Moreover, for all the four species, observed encounter frequencies for either low or high probability samples were generally within or extremely close to the 95% confidence interval for predicted probability of encounter (Figure 1). Therefore, the geostatistical GLMMs of the four above mentioned species passed the validation test.

We fitted a binomial GAM for eight of the study species: (1) greater amberjack (*Seriola dumerili*); (2) almaco jack (*Seriola rivoliana*); (3) Warsaw grouper (*Hyporthodus nigritus*); (4) black grouper (*Mycteroperca bonaci*); (5) mutton snapper (*Lutjanus analis*); (6) black drum (*Pogonias cromis*); (7) red drum (*Sciaenops ocellatus*); and (8) sheepshead (*Archosargus probatocephalus*). The binomial GAMs of the eight species had a median AUC value ranging between 0.803 and 0.989 and a median adjusted *R*^2^ value ranging between 0.11 and 0.69 (Table 2). Therefore, the GAMs of the eight above mentioned species passed the validation test.

**Table 1. Diagnostics generated during the estimation of the parameters of the geostatistical generalized linear mixed models fitted in the present study.** *ε* = spatially correlated variability in probability of encounter; *H* = linear transformation representing geometric anisotropy in *ε*; *κ* = range parameter, which governs the distance over which the covariance of *ε* drops to 10% of its pointwise value; *σ_ε_* = standard deviation of *ε*.

| **Species** | **Parameter** | **Lower bound** | **Maximum likelihood estimate (MLE)** | **Upper bound** | **Final gradient** |
| --- | --- | --- | --- | --- | --- |
| Yellowedge grouper | *ln*(*H*) | -50 | 0.06204 | 50 | -2.625.10^-5^ |
|  | *ln*(*H*) | -50 | -0.4866 | 50 | -6.987.10^-5^ |
|  | *σ_ε_* | -50 | -9.979 | 50 | -1.645.10^-5^ |
|  | *log*(*κ*) | -6.356 | -4.851 | 0.2563 | 1.98.10^-4^ |
| Scamp | *ln*(*H*) | -50 | 0.07591 | 50 | -2.204.10^-10^ |
|  | *ln*(*H*) | -50 | -0.2904 | 50 | -9.601.10^-11^ |
|  | *σ_ε_* | -50 | 5.551 | 50 | -8.178.10^-12^ |
|  | *log*(*κ*) | -6.291 | -4.553 | 0.4319 | -1.51.10^-10^ |
| Gag | *ln*(*H*) | -50 | 0.001236 | 50 | 9.545.10^-6^ |
|  | *ln*(*H*) | -50 | -0.6955 | 50 | -1.704.10^-5^ |
|  | *σ_ε_* | -50 | -6.502 | 50 | -5.109.10^-6^ |
|  | *log*(*κ*) | -6.32 | -5.478 | 0.0438 | 4.329.10^-5^ |
| Southern flounder | *ln*(*H*) | -50 | 0.7919 | 50 | -7.604.10^-5^ |
|  | *ln*(*H*) | -50 | 1.171 | 50 | -0.00021 |
|  | *σ_ε_* | -50 | 5.732 | 50 | -3.716.10^-5^ |
|  | *log*(*κ*) | -6.321 | -5.443 | 1.018 | -0.0003546 |

**Table 2. Evaluation of the binomial generalized additive models (GAMs) fitted in the present study.** The figures provided are medians and 95% confidence intervals (within square brackets). AUC = area under the receiver operating characteristic curve – adj-R^2^ = adjusted coefficient of determination.

| **Species** | **AUC** | **adj-R^2^** |
| --- | --- | --- |
| Greater amberjack | 0.873  [0.855 ; 0.885] | 0.11  [0.10 ; 0.12] |
| Almaco jack | 0.934  [0.891 ; 0.956] | 0.14  [0.13 ; 0.14] |
| Warsaw grouper | 0.876  [0.770 ; 0.927] | 0.11  [0.11 ; 0.12] |
| Black grouper | 0.955  [0.898 ; 0.965] | 0.17  [0.15 ; 0.22] |
| Mutton snapper | 0.989  [0.968 ; 0.995] | 0.69  [0.64 ; 0.74] |
| Black drum | 0.891  [0.886 ; 0.894] | 0.11  [0.11 ; 0.12] |
| Red drum | 0.848  [0.844 ; 0.854] | 0.16  [0.14 ; 0.16] |
| Sheepshead | 0.904  [0.896 ; 0.912] | 0.32  [0.30 ; 0.34] |

**Figure 1. Predicted probability of enconter (gray line: mean; shading: 95% confidence interval) versus observed encounter frequency (black dots) for yellowedge grouper (*Hyporthodus flavolimbatus*), scamp (*Mycteroperca phenax*), gag (*Mycteroperca microlepis*) and southern flounder (*Paralichthys lethostigma*).**

**
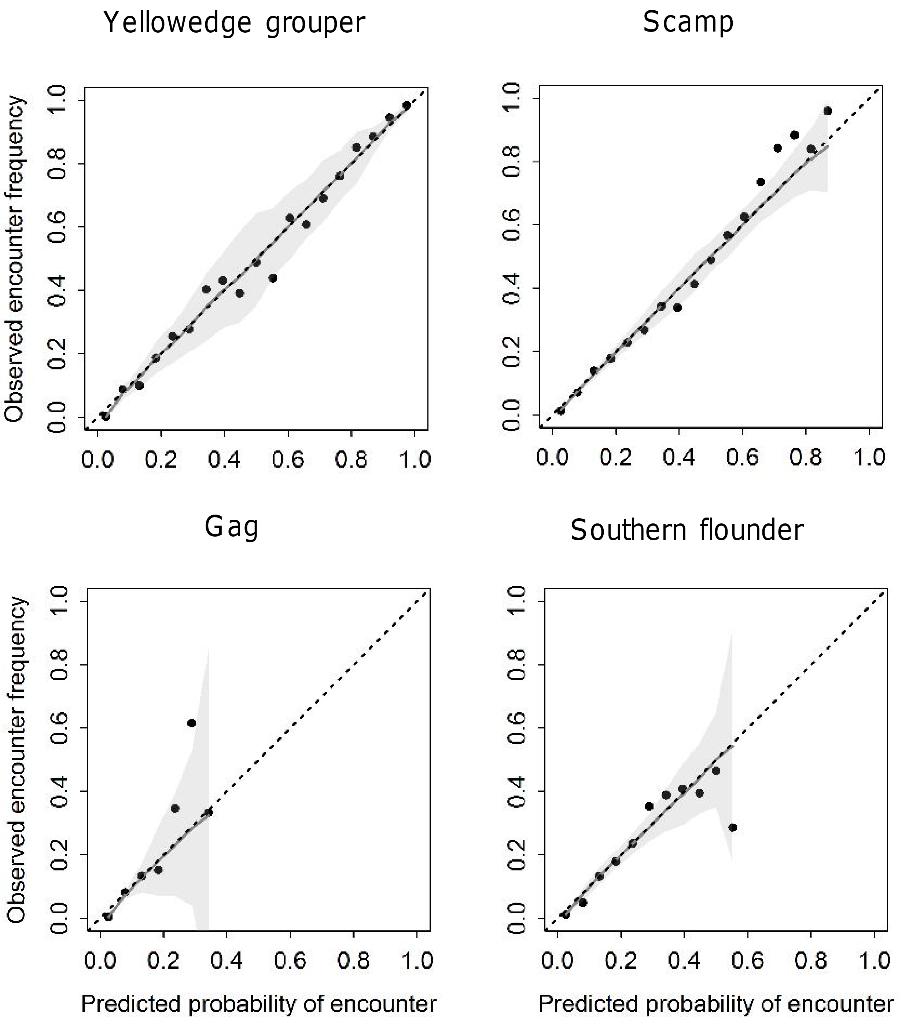
**

**Supplementary Table S1. Summary of the species distribution models (SDMs) developed in the present study.** These SDMs were fitted to data from the large monitoring database for the U.S. Gulf of Mexico (GOM), which gathers all the fisheries-independent and fisheries-dependent data that were collected in the U.S. GOM over the period 2000-2016 using random sampling methods. Details about the different fisheries-independent and fisheries-dependent monitoring programs included in the large monitoring database for the U.S. GOM and considered in this study can be found in Supplementary Table S2. GLMM = generalized linear mixed model – GAM = generalized additive model.

| **Species** | **Monitoring data available for the species** | **SDM fitted for the species** | **Monitoring data used to fit a SDM for the species** | **Sampling years considered to fit a SDM for the species** |
| --- | --- | --- | --- | --- |
| Greater amberjack (*Seriola dumerili*) | OBSLL (204 encounter estimates), OBSVL (122), FLOBS (95), VIDEO (72), SBLOP (56), BLL (3), EASALL (3), PCVIDEO (3), EASAVL (2), LAVL (2), VL (1) | GAM | OBSLL, OBSVL, FLOBS, VIDEO, SBLOP | 2002-2007, 2009-2016 |
| Almaco jack (*Seriola rivoliana*) | OBSLL (75 encounter estimates), OBSVL (39), VIDEO (9) | GAM | OBSVL, OBSLL, VIDEO | 2008, 2010-2015 |
| Yellowedge grouper (*Hyporthodus flavolimbatus*) | OBSLL (1575 encounter estimates), SBLOP (310), BLL (182), OBSVL (149), EASALL (92), INBLL (13), EASAVL (4), VIDEO (4), SMALLPEL (2), POP (1), TRAWL (1), VL (1) | Geostatistical GLMM | OBSLL, SBLOP, BLL, OBSVL, EASALL | 2001-2015 |
| Warsaw grouper (*Hyporthodus nigritus*) | OBSLL (34 encounter estimates), OBSVL (18), SBLOP (3), EASALL (2), EASAVL (1), VIDEO (1) | GAM | OBSLL, OBSVL, SBLOP | 2009-2010, 2014 |
| Scamp (*Mycteroperca phenax*) | OBSLL (1483 encounter estimates), OBSVL (1180), SBLOP (335), VIDEO (221), FLOBS (72), PCVIDEO (35), EASAVL (15), TRAWL (9), VL (7), PCTRAP (6), LAVL (4), FLTRAP (2) | Geostatistical GLMM | OBSLL, OBSVL, SBLOP, VIDEO, FLOBS | 2007-2015 |
| Gag (*Mycteroperca microlepis*) | OBSLL (182 encounter estimates), OBSVL (82), SBLOP (71), BLL (6), FLOBS (3), MSHAND (1), VIDEO (1) | Geostatistical GLMM | OBSLL, OBSVL, SBLOP | 2007-2015 |
| Black grouper (*Mycteroperca bonaci*) | OBSLL (93 encounter estimates), SBLOP (50), OBSVL (13) | GAM | OBSVL, FLOBS, OBSLL | 2007, 2009-2015 |
| Yellowmouth grouper (*Mycteroperca interstitialis*) | OBSLL (11 encounter estimates), OBSVL (8), VIDEO (8), BLL (1), EASALL (1), EASAVL (1), LAVL (1), SBLOP (1), VL (1) | None, due a lack of monitoring data for the species | - | - |
| Yellowfin grouper (*Mycteroperca venenosa*) | None | None, due to an absence of data for the species | - | - |
| Nassau grouper (*Epinephelus striatus*) | None | None, due to an absence of data for the species | - | - |
| Goliath grouper (*Epinephelus itajara*) | TRAWL (4 encounter estimates), OBSVL (2), BLL (1), FLBAY (1), SBLOP (1), VIDEO (1) | None, due a lack of monitoring data for the species | - | - |
| Mutton snapper (*Lutjanus analis*) | OBSLL (229 encounter estimates), SBLOP (45), OBSVL (14), FLOBS (14), FLHAUL (8), VIDEO (8), BLL (3), TRAWL (3), EASALL (1), FLBAY (1) | GAM | OBSLL, SBLOP, OBSVL | 2006-2007, 2009-2013, 2015 |
| Cubera snapper (*Lutjanus cyanopterus*) | OBSLL (4 encounter estimates), OBSVL (2) | None, due a lack of monitoring data for the species | - | - |
| Black drum (*Pogonias cromis*) | TXGILL (768 encounter estimates), FLHAUL (148), TXTRAWL (87), INBLL (22), TRAWL (18), ALGILL (12), MSHAND (7), MSGILL (6), BLL (5), FLTRAWL (4), FLBAY (2), OBSGILL (2), TXBLL (2), GULFSPAN (1), LAVL (1), TXSEINE (1), VL (1) | GAM | TXGILL, FLHAUL, TXTRAWL, TRAWL, INBLL, ALGILL | 2000-2015 |
| Red drum (*Sciaenops ocellatus*) | TXGILL (1583 encounter estimates), FLHAUL (156), INBLL (67), TRAWL (49), BLL (38), MSHAND (21), ALGILL (16), TXBLL (14), TXTRAWL (10), EASALL (9), MSGILL (9), LAVL (5), VL (5), OBSVL (4), OBSGILL (3), TXSEINE (3), FLBAY (1), FLOBS (1), GULFSPAN (1), OBSLL (1), POP (1) | GAM | TXGILL, FLHAUL, INBLL, TRAWL, BLL, MSHAND, ALGILL, | 2000-2015 |
| Sheepshead (*Archosargus probatocephalus*) | TXGILL (898 encounter estimates), FLHAUL (763), TXTRAWL (165), ALGILL (64), TXSEINE (37), OBSVL (30), FLTRAWL (12), FLBAY (9), FLOBS (4), GULFSPAN (4), TRAWL (4), OBSGILL (3), FLPURSE (2), MSHAND (1) | GAM | TXGILL, FLHAUL, TXTRAWL, ALGILL | 2000-2015 |
| Southern flounder (*Paralichthys lethostigma*) | TXGILL (953 encounter estimates), TRAWL (48), SMALLPEL (38), ALGILL (32), TXSEINE (23), FLHAUL (12), TXTRAWL (11), FLTRAWL (5), OBSLL (3), OBSSHRIMP (2), GULFSPAN (1), OBSVL (1), SBLOP (1) | Geostatistical GLMM | TXGILL, TRAWL, SMALLPEL, ALGILL | 2000-2015 |

**Supplementary Table S2. Monitoring programs from the large monitoring database for the U.S. Gulf of Mexico (GOM) considered in the present study.** FL = Florida – AL = Alabama – MS = Mississippi – LA = Louisiana – TX = Texas – NC = North Carolina - WFS = West Florida Shelf – EEZ = Exclusive Economic Zone - NMFS = National Marine Fisheries Service – NOAA = National Oceanic and Atmospheric Administration – SEFSC = Southeast Fisheries Science Center – GSMFC = Gulf States Marine Fisheries Commission – FWC = Florida Fish and Wildlife Conservation Commission - FWRI = Fish and Wildlife Research Institute – LDWF = Louisiana Department of Wildlife and Fisheries – NSU = Nova Southeastern University – FSU = Florida State University – DISL = Dauphin Island Sea Laboratory – USM = University of Southern Mississippi – LSU = Louisiana State University – SEAMAP = Southeast Area Monitoring and Assessment Program – NRDA = Natural Resource Damage Assessment.

| **Name of the monitoring program** | **Type of monitoring program (fisheries-independent, fisheries-dependent)** | **Type of institution in charge of the monitoring program (Federal, State, other)** | **Name of the institution in charge of the monitoring program** | **Sampling locations** | **Seasons of data collection** | **Areas sampled** | **Years for which we have data** | **Sampling effort** | **Comments** |
| --- | --- | --- | --- | --- | --- | --- | --- | --- | --- |
| Alabama Marine Resources Division (AMRD) Fisheries Assessment and Monitoring Program (FAMP) Gillnet Survey (Alias: ALGILL) | Fisheries-independent | State | AMRD, Dauphin Island, AL | ● Stratified random stations throughout MS Sound, Mobile Bay, and Perdido System | Year-round | AL | 2000 – 2015 | Number per net | ● Small and large mesh nets used to allow biologist to account for the varying sizes and shapes of finfish inhabiting the coastal waters of Alabama. The small mesh net consists of 5 panels of net in varying stretched mesh sizes (2"-4" mesh). The large mesh net consists of 4 panels of net in varying stretched mesh sizes (4.5"-6" mesh).  ● Otoliths are collected from several species.  ● Environmental parameters recorded: temperature, salinity, dissolved oxygen concentration, and depth of water by midpoint of mesh size. |
| NMFS Bottom Longline Survey (Alias: BLL) | Fisheries-independent | Federal | NOAA, SEFSC, Mississippi Laboratories (MSLAB), Pascagoula, MS | ● Randomly chosen locations over the entire continental shelf of the U.S. GOM | Summer (July – September) | FL – TX | 2000-2014 | Number per 100 hook hours | ● Species targeted: coastal sharks, snappers, and groupers.  ● Many changes to sampling protocols (e.g., target species, hook types, spatial coverage) have complicated reliability of derived time series. |
| Deep Pelagic Nekton Dynamics of the GOM (DEEPEND) Survey (Alias: DEEPEND) | Fisheries-independent | Federal | NSU, Dania Beach, FL | ● Oceanic GOM from the surface to 1500 m in the region of the *Deepwater Horizon* oil spill | May and August | LA | 2015-2016 | Number per tow | ● Midwater trawling conducted using a 10-m² mouth area MOCNESS midwater trawl. This is the same depth scheme as that used during the NOAA NRDA Offshore Nekton Sampling and Analysis Program.  ● Environmental parameters recorded: maximum water column depth, minimum and maximum temperature, minimum and maximum salinity. |
| NMFS Expanded Annual Stock Assessment (EASA) Survey – Longline (Alias: EASALL) | Fisheries-independent | Federal | NOAA, SEFSC, MSLAB, Pascagoula, MS | ● Randomly chosen locations over the entire continental shelf of the U.S. GOM between 9 and 400 m | Spring – fall (April – October) | FL – TX | 2011 | Number per hook hour | ● EASA: congressional supplemental sampling program that contracted commercial vessels and outfitted them with contracted biologists. The EASA survey used bottom longline and vertical line gears to sample species of the reef fish complex.  ● Sampling took place during daylight hours and used the same gear and sample methodology as the NMFS bottom longline survey. |
| NMFS EASA Survey - Vertical Line (Alias: EASAVL) | Fisheries-independent | Federal | NOAA, SEFSC, MSLAB, Pascagoula, Mississippi | ● Randomly chosen locations over the entire continental shelf of the U.S. GOM between 9 and 400 m | Spring – fall (April – October) | FL – TX | 2011 | Number per hook hour | ● Vertical line gear used to target high-relief hard-bottom habitat inaccessible to longline or trawls.  ● Some stations fished vertical lines independently; others fished vertical lines simultaneously with longlines. |
| Fish and Wildlife Research Institute (FWRI) Bay Seine Survey (Alias: FLBAY) | Fisheries-independent | State | FWC, FWRI, St. Petersburg, FL | ● Major estuaries on FL west coast | Year-round | FL | 2000-2015 | Number per 100 m^2^ of area sampled | ● Employs a 21.3-m, 1.8-m deep center bag seine to target young-of-the-year (YOY) and juvenile fishes in shallow waters (<1.8 m).  ● Samples areas where the water depth is less than 1.5 m, such as tidal flats, mangrove fringes, sea wall habitats, sloping beaches, and banks.  ● Environmental parameters recorded: tide, wind direction, wind speed, cloud cover, precipitation (yes/no), bottom type (sponge, rocks, oysters, etc.), bottom vegetation, water conductivity, depth, water pH, dissolved oxygen concentration, water salinity, water temperature, percentage of the bottom covered by any type of bottom vegetation, distance to mean high tide mark, and distance to land-water interface. |
| FWRI Haul Seine Survey (Alias: FLHAUL) | Fisheries-independent | State | FWC, FWRI, St. Petersburg, FL | ● Major estuaries on FL west coast | Year-round | FL | 2000-2015 | Number per haul | ● Employs a 183-m center-bag haul seine to sample large-bodied fish along shoreline habitats in waters <2.5 m deep.  ● Environmental parameters recorded: tide, wind direction, wind speed, cloud cover, precipitation (yes/no), bottom type (sponge, rocks, oysters, etc.), bottom vegetation, water conductivity, depth, water pH, dissolved oxygen concentration, water salinity, water temperature, percentage of the bottom covered by any type of bottom vegetation, distance to mean high tide mark, and distance to land-water interface. |
| FWRI For-Hire At-Sea Observer Program (Alias: FLOBS) | Fisheries-dependent | State | FWC, FWRI, St. Petersburg, FL | ● Randomly sample trips from a list of cooperative charter, headboat, and multi-day headboat vessels operating off the west coast of FL and in the Florida Keys | Year-round | FL | 2009 – 2016 | Number of fish caught (harvested and/or discarded) per trip/station/angler/rod/hour | ● Collects detailed information on recreational charter and headboat catch, particularly for discarded fish.  ● Vessels are randomly selected each month throughout the year, stratified by vessel type (charter, headboat, multi-day) and region (Panhandle, western peninsula, keys). Biologists board selected vessels with permission from the captain and observe anglers as they fish on the recreational trip, record species/size/gear/disposition/release condition, and record station depth and latitude/longitude (degrees/minutes). |
| FWRI Purse Seine Survey (Alias: FLPURSE) | Fisheries-independent | State | FWC, FWRI, St. Petersburg, FL | ● Major estuaries on FL west coast | Spring – fall (April – October) | FL | 2000-2004 | Number per haul | ● Employed a 183-m purse seine to collect sub-adult and adult fishes in intermediate depths (1-3.3m).  ● Environmental parameters recorded: tide, wind direction, wind speed, cloud cover, precipitation (yes/no), bottom type (sponge, rocks, oysters, etc.), bottom vegetation, water conductivity, depth, water pH, dissolved oxygen concentration, water salinity, water temperature, percentage of the bottom covered by any type of bottom vegetation, distance to mean high tide mark, and distance to land-water interface. |
| FWRI Reef Fish Trap Survey (Alias: FLTRAP) | Fisheries-independent | State | FWC, FWRI, St. Petersburg, FL | ● Randomly chosen sampling stations on WFS between 26° and 28°N and depths from 10-110 m.  ● If no reef habitat identified in sampling unit by a three-pass acoustic survey, sampling effort relocated to a nearby sampling unit with reef habitat | Summer - fall (June – October) | FL | 2010-2014 | Number per trap set | ● Employs chevron traps following the same protocol as NMFS.  ● Sampling universe during 2008 considered an experiment because a high proportion of sampling effort occurred at sites with no reef habitat.  ● Some inconsistencies between day and night sampling.  ● Environmental parameters recorded: wind direction, wind speed, cloud coverage, precipitation, and sea state. |
| FWRI Trawl Survey (Alias: FLTRAWL) | Fisheries-independent | State | FWC, FWRI, St. Petersburg, FL | ● Major estuaries on FL west coast | Year-round | FL | 2000-2015 | Number per 100 m^2^ of area sampled | ● Employs a 6.1-m otter trawl to sample demersal habitats in waters 1.8 - 7.6 m deep.  ● Environmental parameters recorded: tide, wind direction, wind speed, cloud cover, precipitation (yes/no), bottom type (sponge, rocks, oysters, etc.), bottom vegetation, water conductivity, depth, water pH, dissolved oxygen concentration, water salinity, water temperature, percentage of the bottom covered by any type of bottom vegetation, distance to mean high tide mark, and distance to land-water interface. |
| Gulf of Mexico Fisheries Information Network (GulfFIN) Head Boat Port Sampling (Alias: GULFFINPORT) | Fisheries-dependent | Federal | NOAA Beaufort Laboratory, NC SEFSC, Miami, FL | ● Dockside intercepts from hook and line fishing | Year-round | GOM Exclusive Economic Zone - TX, LA, MS, AL, FL | 2014-2015 | Marine recreational catch per unit effort | ● Parameters collected: logbook census program for federally permitted headboats and dockside sampling program to gather landings, angler effort, biological data (species, length, weight), and biological structures (otoliths and gonads).  ● The geographic coordinates associated with some of the GULFFINPORT data are located inland (due to fishers unwilling to share the geographic coordinates of their fishing locations). |
| NMFS Gulf of Mexico Shark Pupping and Nursery (GULFSPAN) Survey (Alias: GULFSPAN) | Fisheries-independent | Federal | NOAA, SEFSC, PCLAB, Panama City, FL and others (see comments) | ● Coastal areas of the GOM | Spring – fall (March – October) | FL – LA | 2002-2015 | Number per net hour | ● Collaborative effort between NMFS Panama City Lab, UM, FSU, DISL, USM, and LSU. Fisheries-independent survey to examine distribution and abundance of juvenile sharks in GOM coastal areas.  ● Employs a gillnet, consisting of six panels 3.0 m (10 ft) deep and 30.5 m (100 ft) long. Panels are strung together and fished as a single gear. A range of gear specifications have been used. |
| SEAMAP GOM Inshore Bottom Longline Survey (Alias: INBLL) | Fisheries-independent | Federal | NMFS, GSMFC, Ocean Spring, MS | ● Randomly chosen locations over TX, LA, MS, AL shallow waters (3-10 m) | Spring (April-May), summer (June-July), and fall (August-September) | North GOM - TX, LA, MS, and AL shallow waters (3-10 m) | 2008 - 2015 | Number per 100 hooks hour | ● Complements an existing long-term fisheries independent survey currently being conducted by NOAA Fisheries, by targeting shark and finfish species within the shallow waters of the north central GOM.  ● Objectives of the survey: collect information on coastal shark and finfish abundances and distribution with a 1-mile longline and collect environmental data.  ● One hundred gangions and hooks are attached equal distance along the 1-mile longline. The hooks soak for one hour.  ● Environmental parameters recorded: turbidity, bottom depth, temperature at the surface, temperature at mid depth, temperature at the bottom, salinity at the surface, salinity at mid depth, salinity at the bottom, dissolved oxygen concentration at the surface, dissolved oxygen concentration at mid depth, and dissolved oxygen concentration at the bottom.  ● Over the period 2011-2014, LDWF sampled waters >10 m depth between 89°W to 91°W. Beginning in 2015, LDWF extended sampling from 89°W to 94°W and limited sampling within the 3-10 m depth range. |
| Louisiana Department of Wildlife and Fisheries (LDWF) Vertical Line Survey (Alias: LAVL) | Fisheries-independent | State | LDWF, Baton Rouge, LA | ● Near platforms, oil rigs, natural bottom structures, and natural and artificial reefs off the LA coast between 60 to 360 feet in depth | Spring – fall (May – October) | LA | 2011 – 2016 | Number per set | ● Targets red snapper (*Lutjanus campechanus*), groupers, amberjacks, and triggerfish.  ● Employs 3 lines with 10 hooks each, with each line having a different size hook.  ● Environmental parameters recorded: water quality, water temperature, salinity, dissolved oxygen concentration, depth, sea condition (Beaufort scale), cloud cover, wind speed, and wave height. |
| Mississippi Department of Marine Resources (MDMR) Sport Fish Shark Gillnet Survey (Alias: MSGILL) | Fisheries-independent | State | USM Gulf Coast Research Laboratory, Ocean Springs, MS | ● Locations in the MS Sound in close proximity to barrier islands | Spring – fall (March – October) | MS | 2004-2016 | Number per 100 gillnets per hour | ● Sampling conducted with a 152.4 x 3 m gillnet consisting of five 30.5-meter panels of 4.5, 5.1, 5.7, 6.4, and 7.0 cm square mesh.  ● Only elasmobranchs are measured and teleosts were documented by abundance (number) caught in each panel in later years only. |
| MDMR Sport Fish Shark Handline Survey (Alias: MSHAND) | Fisheries-independent | State | USM Gulf Coast Research Laboratory, Ocean Springs, MS | ● Locations in the MS Sound in close proximity to barrier islands | Spring – fall (March – October) | MS | 2004-2016 | Number per 100 hooks per hour | ● The handline is 500 ft, has 50 gangions with 12/0 hooks and is soaked for an hour.  ● Length measurements were performed in later years only. |
| MDMR Fisheries Assessment and Monitoring (FAM) Trawl Survey (Alias: MSTRAWL) | Fisheries-independent | State | USM Gulf Coast Research Laboratory, Ocean Springs, MS | ● Random stations in MS territorial waters | Year-round | MS | 2009-2015 | Number per tow | ● The MSTRAWL monitoring program is relatively unique in that it conducts sampling at both fixed and random stations; in the present study, we considered only the random stations sampled by the MSTRAWL monitoring program.  ● Employs a 16 ft flat trawl for 10 minutes at each station.  ● Does not target any specific species.  ● Environmental parameters recorded: depth, wind speed, wind direction, transparency, sea state, cloud cover, habitat type (sand, mud, clay, marsh submerged vegetation, etc.), bottom temperature, surface temperature, bottom salinity, surface salinity, bottom dissolved oxygen concentration, and surface dissolved oxygen concentration. |
| NMFS Southeast Gillnet Observer Program (Alias: OBSGILL) | Fisheries-dependent | Federal | NOAA, SEFSC, PCLAB, Panama City, FL | ● Trained observers randomly placed on vessels throughout the GOM | Year-round | FL – LA | 2000-2002, 2006, 2007-2009, 2010-2016 | Number per set | ● Observer program primarily designed to monitor all vessels that have an active directed shark permit and fish with gillnet gear, including all anchored (sink, stab, set), strike, or drift gillnets.  ● Some of the OBSGILL data are collected in very close proximity (using different panels of the same gear).  ● Environmental parameters recorded: depth, bottom type (sand, mud, etc.), wind direction, wind speed, wave height, presence/absence of seabirds and seabird species present, and presence/absence of dolphins and dolphin species present. |
| Reef Fish Bottom Longline Observer Program (Alias: OBSLL) | Fisheries-dependent | Federal | NOAA, SEFSC, Galveston Laboratory, Galveston, TX | ● NMFS observers placed on reef fish vessels operating throughout the GOM based on randomized selection stratified by season, gear, and region  ● Proportional sampling used to direct coverage levels toward region and gear strata with higher levels of fishing effort, while continuing to sample strata with lower fishing effort | Year-round | FL – TX | 2006 – 2016 | Number caught per 100 hook hours | ● Mandatory observer program implemented in 2006 to characterize the commercial reef fishery operating in the U.S. GOM. The OBSLL dataset covers the same set of data as the commercial longline and although this set contains a more accurate description of effort, it is a much shorter time series.  ● Environmental parameters recorded: bottom depth. |
| Southeastern Shrimp Fisheries Observer Coverage (Alias: OBSSHRIMP) | Fisheries-dependent | Federal | NOAA, SEFSC, Galveston Laboratory, Galveston, TX | ● NMFS-approved observers placed on randomly selected shrimp vessels targeting either penaeid or rock shrimps | Year-round | FL – TX | 2000-2016 | Number per tow | ● Vessel, gear and biological data collected by fishery observers from the GOM and South Atlantic commercial shrimp fishery. Finfish bycatch includes red snapper, groundfish, with Atlantic croaker (*Micropogonias undulatus*) and longspine porgy (*Stenotomus caprinus*) being the dominant species both in number and by weight for the GOM.  ● Length is recorded for a few species only.  ● Environmental parameters recorded: bottom depth. |
| Reef Fish Bottom Vertical Line Observer Program (Alias: OBSVL) | Fisheries-dependent | Federal | NOAA, SEFSC, Galveston Laboratory, Galveston, TX | ● NMFS observers placed on reef fish vessels operating throughout the GOM based on randomized selection stratified by season, gear, and region  ● Proportional sampling used to direct coverage levels toward region and gear strata with higher levels of fishing effort, while continuing to sample strata with lower fishing effort | Year-round | FL – TX | 2006-2016 | Number caught per hook hour | ● Mandatory observer program implemented in 2006 to characterize the commercial reef fishery operating in the U.S. GOM. The gear types assessed include bandit reel and handline.  ● Environmental parameters recorded: bottom depth. |
| NMFS Panama City Trap Survey (Alias: PCTRAP) | Fisheries-independent | Federal | NOAA, SEFSC, Panama City Laboratory (PCLAB), Panama City, FL | ● Randomly chosen natural reefs sites on the northern half of the WFS | Spring – fall (May – October) | Northwest FL | 2004-2014 | Catch per trap hour | ● Employs chevron traps identical to those used in the Marine Resources Monitoring Assessment and Prediction (MARMAP) program, with the exception of a 50% smaller opening in the PCTRAP monitoring program.  ● Change in survey sampling design in 2009. |
| NMFS Panama City Video Survey (Alias: PCVIDEO) | Fisheries-independent | Federal | NOAA, SEFSC, PCLAB, Panama City, FL | ● Randomly chosen natural reefs sites on the northern half of the WFS | Spring – fall (May – October) | Northwest FL | 2005 – 2013 | MinN | ● Target species: snappers, groupers, Gray Triggerfish (*Balistes capriscus*), red porgy (*Pagrus pagrus*), white grunt (*Haemulon plumieri*), black sea bass (*Centropristis striata*), hogfish (*Lachnolaimus maximus*), and amberjacks.  ● High definition digital video cameras used between 2005 and 2008, and stereo imaging system used from 2009 onward.  ● Length measurements started in 2009. Body length is estimated for a subset of species recorded by cameras, generally only exploited species. Body lengths are measured in a manner similar to the “min count” procedure, but not necessarily at that exact time to allow for the maximum number of measurements to be taken while ensuring no over counting. |
| NMFS Pelagic Observer Program (Alias: POP) | Fisheries-dependent | Federal | NMFS, SEFSC, Miami Laboratory, Miami, FL | ● Trained observers randomly placed on vessels throughout the GOM | Year-round | FL – LA | 2000-2015 | Number caught per hook | ● Monitors a mobile U.S. pelagic longline fleet ranging from Newfoundland along the Western Atlantic to Brazil and throughout the GOM. Vessels range in size from 35 to 90 feet. |
| NMFS Shark Bottom Longline Observer Program (Alias: SBLOP) | Fisheries-dependent | Federal | NOAA, SEFSC, PCLAB, Panama City, FL | ● Trained observers randomly placed on vessels throughout the GOM | Year-round | FL – LA | 2005 – 2015 | Number caught per 10,000 hooks | ● Observer program primarily designed to monitor shark fisheries but also other bottom longline fisheries including deep and shallow water grouper and tilefish fisheries in the GOM.  ● Regional differences in longline characteristics exist and longlines typically extend 5−15 miles and consist of 500−1500 hooks.  ● Environmental parameters recorded: water surface temperature, and bottom depth. |
| NMFS Small Pelagics Survey (Alias: SMALLPEL) | Fisheries-independent | Federal | NOAA, SEFSC, MSLAB, Pascagoula, MS | ● Outer shelf and upper slope survey (between 110 and 500 m station depth) of the U.S. GOM, with stations selected with a proportional allocation based on stratum area | Fall – winter | FL – TX | 2002 – 2014 | Number per trawl-hour | ● Employs a 27.4 m high-opening fish trawl towed for approximately 30 minutes.  ● By 2004, the SMALLPEL monitoring program became a mid to outer shelf and upper slope survey (i.e. between 50 and 500 m station depth) to overlap with the SEAMAP trawl/groundfish monitoring program. The SMALLPEL monitoring program provides range extensions for species occurring in the SEAMAP trawl/groundfish monitoring program.  ● The acoustic data that the Mississippi Laboratories of NOAA's SEFSC are working up is collected during the SMALLPEL working program (as well as during the SEAMAP Ichthyoplankton monitoring program, which is not included in the large monitoring database for the U.S. GOM). |
| SEAMAP Groundfish Trawl Survey (Alias: TRAWL) | Fisheries-independent | Federal | NMFS, GSMFC, Ocean Spring, MS | ● Stratified random sampling throughout the northern GOM, with station location assignment derived from depth zones, shrimp statistical zones, and time of day | Summer (July – August), fall (September – December), and, sometimes, spring (April – June) and winter (January – March) | AL – TX, FL (since 2008) | 2000-2015 | Number per trawl-hour | ● Employs a semi balloon shrimp trawl (3.8-m footrope).  ● Its design has remained relatively consistent since 1987; however, changes have occurred in station allocation and area surveyed.  ● Also collects larvae as in the SEAMAP Ichthyoplankton monitoring program.  ● Environmental parameters recorded: bottom depth, mid-water depth, surface turbidity, bottom turbidity, mid-water turbidity, precipitation (yes or no), cloud cover, cloud type, bottom temperature, surface temperature, mid-water temperature, bottom salinity, surface salinity, mid-water salinity, bottom chlorophyll-a concentration, surface chlorophyll-a concentration, mid-water chlorophyll-a concentration, bottom oxygen concentration, surface oxygen concentration, and mid-water oxygen concentration. |
| Texas Parks and Wildlife Department (TPWD) Bottom Longline Survey (Alias: TXBLL) | Fisheries-independent | State | TPWD, Port O'Connor, TX | ● Random stratified block design with effort within each block allocated across three depth strata in the Corpus Christi and Galveston Bay regions | Spring – fall (March – October) | TX | 2008 – 2015 | Number per soak hour | ● Sampling protocol and equipment follows the procedures established by the NMFS bottom longline monitoring program.  ● The longline gear consists of a 1.8 km (426 kg test) monofilament mainline and 100, 3.7 m gangions (332 kg test monofilament) outfitted with #15/0 circle hooks and baited with Atlantic mackerel (*Scomber scombrus*). The longline fishes for one hour from the time of last high-flier deployment to the time of first high-flier retrieval. |
| TPWD Gillnet Survey (Alias: TXGILL) | Fisheries-independent | State | TPWD, Port O'Connor, TX | ● Major bay systems along the TX coast  ● Sample locations drawn independently and without replacement for each season | Spring (April – June), fall (September – November) | TX | 2000-2015 | Number captured per hour soak | ● Uses 183 m long monofilament gill nets set perpendicular to shorelines.  ● Targets subadult and adult finfish.  ● Some changes in areas sampled.  ● Gillnets deployed within 1 hour before sunset, fish overnight, and retrieved within 4 h of sunrise the following day. |
| TPWD Seine Survey (Alias: TXSEINE) | Fisheries-independent | State | TPWD, Port O'Connor, TX | ● Stratified cluster sampling design in TX coastal waters | Year-round | TX | 2000-2015 | Number per haul | ● 18.3 m long Gag seines pulled over 0.03 ha (area swept) along the shoreline.  ● Targets juvenile fish and invertebrates.  ● Some changes in areas sampled. |
| TPWD Trawl Survey (Alias: TXTRAWL) | Fisheries-independent | State | TPWD, Port O'Connor, TX | ● Stratified cluster sampling design in TX coastal waters | Year-round | TX | 2000-2015 | Number per tow | ● Uses a 6.1 m wide trawl towed for 10 minutes in open water and targets juvenile and subadult fish and invertebrates.  ● Some changes in areas sampled. |
| SEAMAP Reef Fish Video Survey (Alias: VIDEO) | Fisheries-independent | Federal | NMFS, GSMFC, Ocean Spring, MS | ● Randomly chosen mesophotic reef sites along the shelf edge break of the GOM | Spring – summer (May – August) | FL – TX | 2001-2007, 2010 | MinN | ● Targets fish species associated with topographic features (e.g., reefs, banks, and ledges).  ● Length measurements are not available for all fish observed on video because of procedural challenges of measuring some fish (e.g., not lined up with laser).  ● No continuous time series due to no sampling between 1998 - 2000 and 2003.  ● Environmental parameters recorded: bottom depth, mid-water depth, surface turbidity, bottom turbidity, mid-water turbidity, precipitation (yes or no), cloud cover, cloud type, bottom temperature, surface temperature, mid-water temperature, bottom salinity, surface salinity, mid-water salinity, bottom chlorophyll-a concentration, surface chlorophyll-a concentration, mid-water chlorophyll-a concentration, bottom oxygen concentration, surface oxygen concentration, mid-water oxygen concentration, presence/absence of relief, presence/absence of dunes, local percentage of silts and clays, local percentage of shells and gravels, local percentage of rock, local percentage of artificial material, local percentage of attached epifauna, local percentage of grass, local percentage of sponge, local percentage of unknown sessile invertebrate organisms, local percentage of algae, local percentage of hard coral, local percentage of soft coral, local percentage of sea whips, local percentage of other epibenthic organisms, presence/absence of oil rig, presence/absence of artificial reefs, maximum height of relief visible in the field of view, and overall average height of relief. |
| SEAMAP GOM Vertical Longline Survey (Alias: VLL) | Fisheries-independent | Federal | NMFS, GSMFC, Ocean Spring, MS | ● Randomly chosen locations over LA waters (18-100 m) and artificial reef zone in AL | All seasons | LA (18-110 m), AL (artificial reef zone) | 2010 - 2015 | Number per hook hour | ● Primary purpose: characterizing the spatial and temporal distribution of commercially and recreationally important reef fish species in the coastal waters of GOM states and the adjoining EEZ. Fisheries-independent data characterizing population dynamics of fish assemblages on non-structured and structured bottom habitats (i.e., natural hard bottom and artificial structures) in offshore waters are also obtained.  ● A typical commercial bandit rig is used and holds approximately 500 feet of clear 300 lb. test main line. A 24 ft. backbone (leader) is attached to the terminal end of the main line. Clear 400 lb. test monofilament is used in the construction of the backbone. An approximately ten pound weight is attached to the terminal end of the backbone. The backbone is rigged with ten 18-inch-long gangions at intervals of two feet. All hooks are Mustad 39960D circle hooks, sizes 8/0, 11/0, and 15/0. Each backbone contains 10 gangions of the same hook size. Each bandit rig is randomly assigned a hook size at the first station.  ● Environmental parameters recorded: turbidity, bottom depth, temperature at the surface, temperature at mid depth, temperature at the bottom, salinity at the surface, salinity at mid depth, salinity at the bottom, dissolved oxygen concentration at the surface, dissolved oxygen concentration at mid depth, dissolved oxygen concentration at the bottom, and bottom type (natural bottom, artificial reef, petroleum platform, pipe). |

**Supplementary Figure S1. Maps showing the probability of encounter and the potential spawning areas of twelve species of the U.S. Gulf of Mexico forming transient spawning aggregations.** The maps shown here were produced from the predictions of geostatistical generalized linear mixed models, or from the predictions of generalized additive models accounting for spatial structure at a broad spatial scale. Depth contours are provided in the right panels and are labeled in 20−, 40−, 60−, 100−, 200−, and 1000−m contours. Figure produced with MATLAB R2017a (<https://www.mathworks.com/products/matlab.html>).


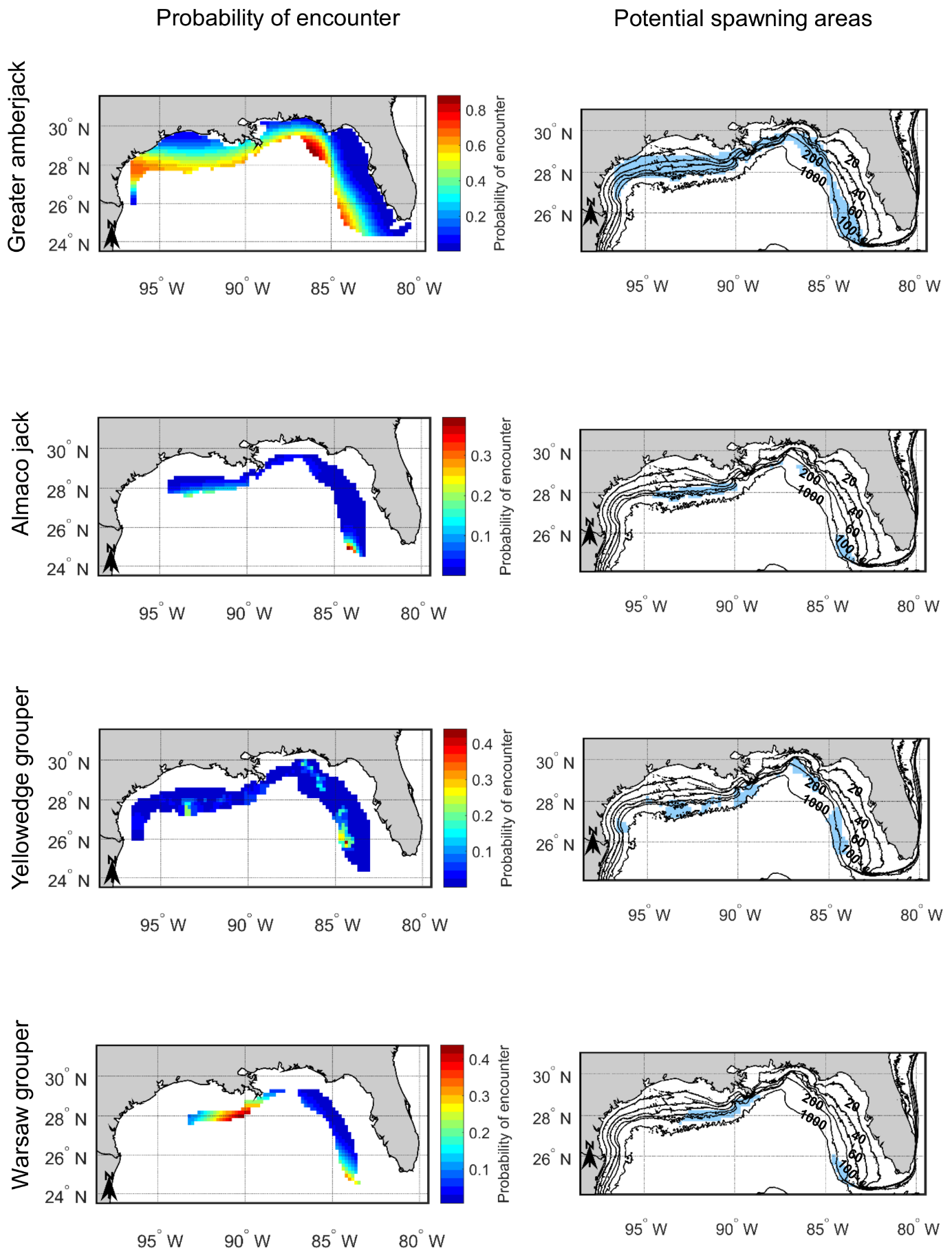


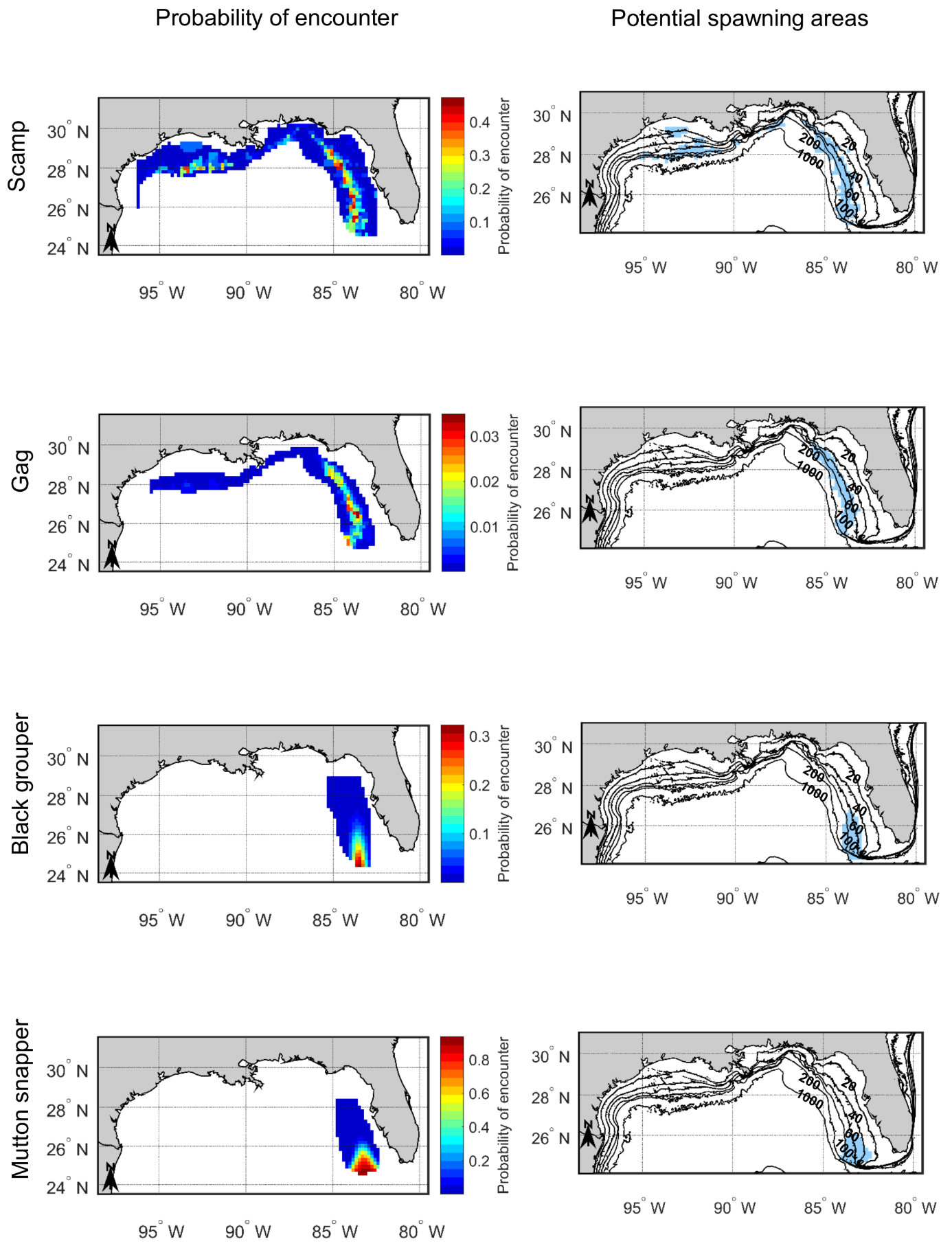


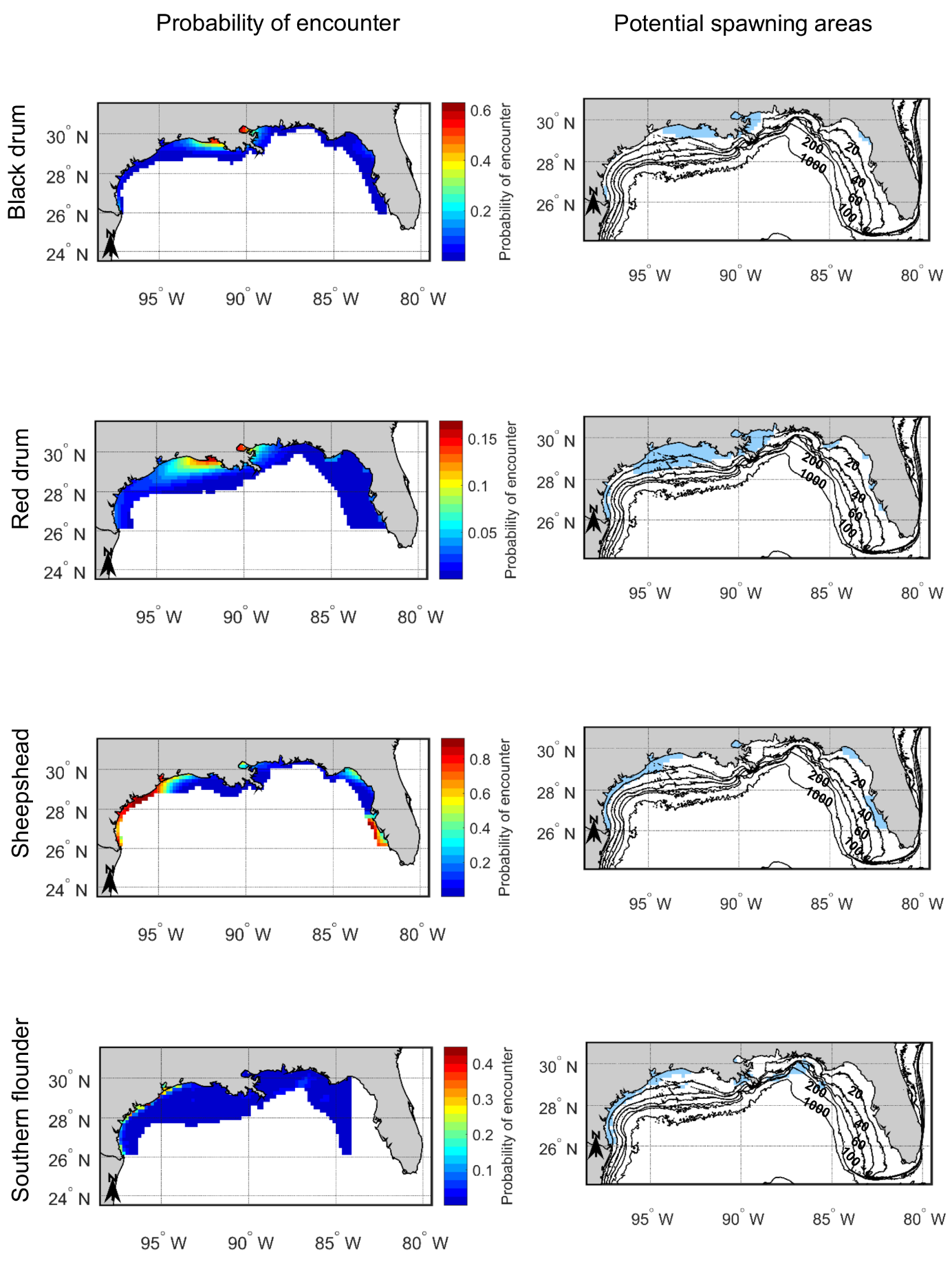

Supplement: Supplementary file 1 — Supplementary Information [file 41598_2018_26898_MOESM1_ESM.docx]
